# Novel Role of the *ALPI* Gene Associated with Constipation Caused by Complement Component 3 Deficiency

**DOI:** 10.3390/ijms25179530

**Published:** 2024-09-02

**Authors:** Hee Jin Song, Ji Eun Kim, Yu Jeong Roh, Ayun Seol, Tae Ryeol Kim, Ki Ho Park, Eun Seo Park, Jin Tae Hong, Sun Il Choi, Dae Youn Hwang

**Affiliations:** 1 Department of Biomaterials Science (BK21 FOUR Program), Life and Industry Convergence Research Institute, College of Natural Resources and Life Science, Pusan National University, Miryang 50463, Republic of Korea; hejin1544@naver.com (H.J.S.); prettyjiunx@naver.com (J.E.K.); buzyu99@naver.com (Y.J.R.); a990609@naver.com (A.S.); xofuf0701@naver.com (T.R.K.); pujihao@naver.com (K.H.P.); geg9393@naver.com (E.S.P.); 2 College of Pharmacy, Chungbuk National University, Chungju 28644, Republic of Korea; jinthong@chungbuk.ac.kr; 3 Henan Key Laboratory of Brain Targeted Bio-Nanomedicine, School of Life Sciences & School of Pharmacy, Henan University, Kaifeng 475004, China; sunil.choi@hotmail.com

**Keywords:** complement C3, constipation, microarray analyses, *ALPI*, acetate

## Abstract

Complement component 3 (C3) deficiency has recently been reported as one of the novel causes of constipation. To identify a unique gene specific to constipation caused by C3 deficiency, the total RNA extracted from the mid colon of C3 knockout (C3 KO) mice was hybridized to oligonucleotide microarrays, and the function of the candidate gene was verified in in vitro and in vivo models. C3 KO mice used for microarrays showed definite phenotypes of constipation. Overall, compared to the wild type (WT), 1237 genes were upregulated, and 1292 genes were downregulated in the C3 KO mice. Of these, the major genes included were lysine (K)-specific demethylase 5D (*KDM5D*), olfactory receptor 870 (*Olfr870*), pancreatic lipase (*PNLIP*), and alkaline phosphatase intestinal (*ALPI*). Specifically, the *ALPI* gene was selected as a novel gene candidate based on alterations during loperamide (Lop)-induced constipation and intestinal bowel disease (IBD). The upregulation of *ALPI* expression treated with acetate recovered the expression level of mucin-related genes in primary epithelial cells of C3 KO mice as well as most phenotypes of constipation in C3 KO mice. These results indicate that *ALPI* plays an important role as the novel gene associated with C3 deficiency-induced constipation.

## 1. Introduction

Complement component 3 (C3) is the most important protein that mediates the complement activation process in the classical, alternative, and lectin pathways for innate immune response [1]. During the activation process, this protein is cleaved into C3a and C3b by C3 convertase [2]. The C3a fragment can mediate various physiological responses including the contraction of smooth muscles, enhancement of vascular permeability, release of histamine from the mast cells, and suppression of B cell polyclonal responses through binding to C3a receptors, while the C3b fragments are combined with C3 convertase to form C5 convertase (C4b2a3b or C3bBb3b), which is responsible for assembly of the membrane attack complex (MAC) [3,4,5]. In addition to the above-mentioned roles, the novel function of C3 has recently been investigated in chronic constipation. Some prominent symptoms of constipation associated with stool excretion, gastrointestinal (GI) transit, mucosal layer thickness, and mucin secretion were detected in sixteen-week-old C3 knockout (C3 KO) mice [6]. These mice also showed dysregulation of the composition of neuronal and interstitial cells of Cajal (ICC), the excitatory and inhibitory transmission of the enteric nervous system (ENS), and expression of C3 receptors in the mid colon during C3 deficiency-induced constipation [7]. Significant inflammatory responses were observed in C3 KO mice with constipation phenotypes through activation of the inducible nitric oxide synthase (iNOS)-mediated cyclooxygenase-2 (COX-2) induction pathway, apoptosis-associated speck-like protein containing a caspase recruitment domain (ASC)-inflammasome pathway and the nuclear factor kappa B (NF-κB) signaling pathway [8]. Furthermore, these mice also showed dysbiosis of fecal microbiota with the presence of *Anaerocolumna*, *Caecibacterium*, *Christensenella*, *Prevotellamassilia*, *Reuthenibacterium* and *Prevotella* populations during constipation [9]. Based on these results, several recent studies carried out to elucidate the novel functions of C3 in the pathogenesis and treatment of constipation have received a lot of attention.

Meanwhile, a limited number of studies have focused on the identification of new genes associated with constipation for the development of novel therapeutics for the treatment of constipation. Around 581 upregulated genes and 216 downregulated genes were detected in the mid colon of Sprague Dawley (SD) rats with constipation induced by loperamide (Lop) during the characterization of the global gene profile in response to the laxative effects of aqueous extracts of *Liriope platyphylla* (AEtLP). They included various functional genes such as solute carrier family 9 (sodium/hydrogen exchanger), member 5 (*SLC9A5*), kallikrein-related peptidase 10 (*KLK10*), fibroblast growth factor 15 (*FGF15*), alkaline phosphatase intestinal (*ALPI*), sphingosine kinase 1 (*SPHK1*), angiotensin I-converting enzyme (peptidyl-dipeptidase A) 1 (*ACE*), SET-binding protein 1 (*SETBP1*), serine (or cysteine) peptidase inhibitor, clade A, member 3N (*SERPINA3N*), lipocalin 2 (*LCN2*) and RAS-like, estrogen-regulated, growth-inhibitor (*RERG*) [10]. Also, in the comparative pharmacokinetics analyses of rhein (a natural botanical medicine), alterations to expression of several genes including *LCN2*, guanylate-binding protein 2 (*GBP2*), indolethylamine N-methyltransferase (*INMT*), glucose-6-phosphatase catalytic subunit (*G6PC*), carbon catabolite repression 4-like (*CCRN41*) and cathedrin17 (*CDH17*) were detected in the liver tissue of constipated SD rats after administration of Lop [11]. Furthermore, genes that were differentially expressed between irritable bowel syndrome (IBS) patients with constipation and healthy controls were detected in the sigmoid colon. Among them, some genes such as T cell differentiation protein (*MAL*), cadherin 16 (*CDH16*), tight junction-associated protein 1 (*TJAP1*), and NADPH oxidase 4 (*NOX4*) were upregulated in the IBS-constipation (IBS-C) group, while other genes such as damage-specific DNA-binding protein 1 (*DDB1*) and CUL4-associated factor 1 (*DCAF1*), aldehyde dehydrogenase 9, family member A1 (*ALDH9A1*), synaptosome-associated protein 47 (*SNAP47*), palladin, cytoskeletal-associated protein (*PALLD*) and NK3 homeobox 1 (*NKX3-1*) were downregulated in the same group [12]. However, no studies to date have attempted to identify the novel genes that play an important role in C3 deficiency-induced constipation despite scientific evidence that C3 deficiency is an important cause of constipation.

In this study, we have attempted to identify a novel gene in response to the C3 deficiency-induced constipation through characterization of changes in the global gene profile. Subsequently, the function of the key gene candidates was further verified by a recovery study in the intestinal epithelial cells of C3 KO mice with constipation phenotypes.

## 2. Results

### 2.1. Verification of C3 Deficiency-Induced Constipation in C3 KO Mice

**Figure 1:**
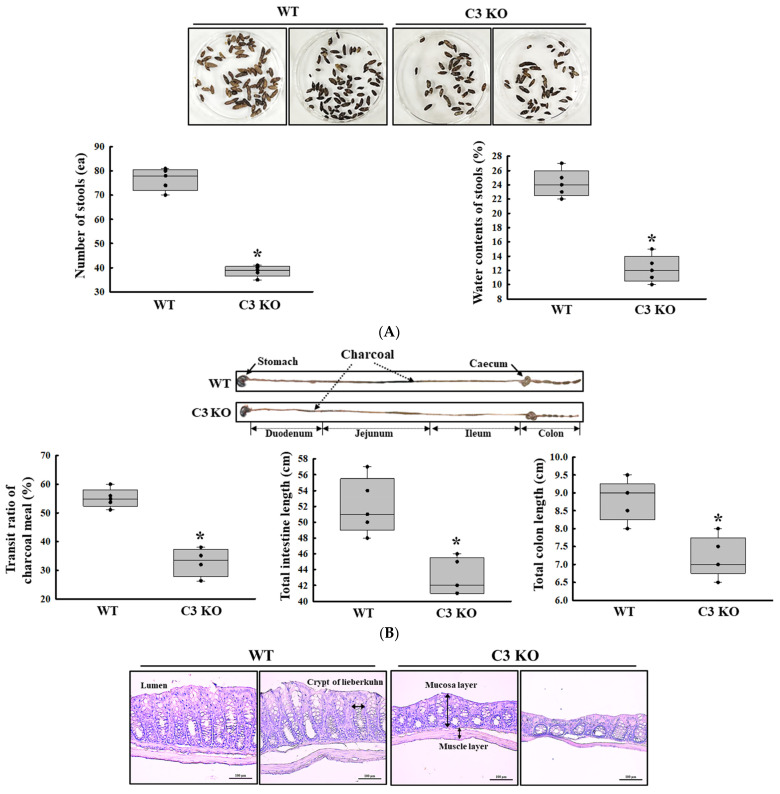

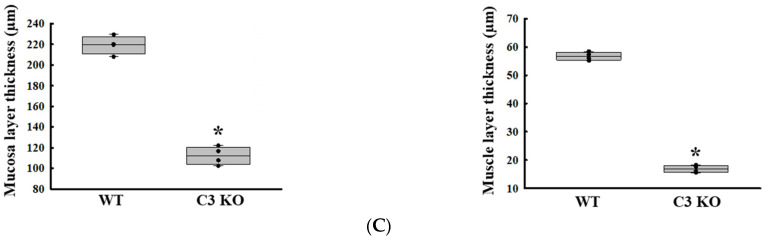
Stool excretion, GI transit ratio and mid colon histology of C3 KO mice. (**A**) Stools excretion. Digital camera images of the stools were taken immediately after collection from the metabolic cage. The total number and water contents of stools were measured as described in the Materials and Methods. Five to six mice per group were used for the stool sample collection, and each parameter was assayed in duplicates. (**B**) GI transit ratio and intestine length. The total intestinal tract was excised from mice of the subset groups treated with charcoal meal powder. The arrows indicate the position of the charcoal meal. Five to six mice per group were used in the GI transit ratio test, and the charcoal meal transit distance, total intestine length and colon length were measured in duplicates. (**C**) Histological structures of the mid colons. The H&E-stained sections of the mid colon from the WT and KO mice were observed at 100× magnification using a light microscope. Five to six mice per group were used in the histological analysis, and each parameter was measured in duplicates in two different slides. The data were presented as the mean ± SD. * *p* < 0.05 vs. WT mice. Abbreviation: WT, wild type; C3 KO, complement C3 knockout; GI, gastrointestinal; H&E, hematoxylin and eosin.

First, we confirmed the constipation phenotypes in C3 KO mice to obtain suitable colon samples for microarray analysis although they have been reported in previous studies. Especially, the verification of constipation phenotypes of mice used in this study is very important to provide a scientific justification for the changes in the global gene expression. To achieve this, the alterations in the stool parameters, GI transit, and the histological structure of mid colon were analyzed in sixteen-week-old wild-type (WT) and C3 KO mice. The number and water content of stools were lower by 52% and 43%, respectively, in the C3 KO mice compared to the WT mice (Figure 1A). A similar pattern was detected in the transit ratio of the charcoal meal and the colon length. These levels were decreased by 29% and 19%, respectively, in the C3 KO mice compared to the WT mice (Figure 1B). Also, the C3 KO mice showed a decrease in mucosal and muscle layer thickness when compared to those of the WT mice (Figure 1C). Therefore, these results suggest that sixteen-week-old C3 KO mice were prominent constipation phenotypes. Furthermore, the above results indicate that these mice with chronic constipation caused by C3 deficiency are suitable for the characterization of the genetic profile.

### 2.2. Characterization of the Changes in the Global Gene Expression Using the Mid Colon Tissue in C3 Deficiency-Induced Constipation

**Figure 2:**
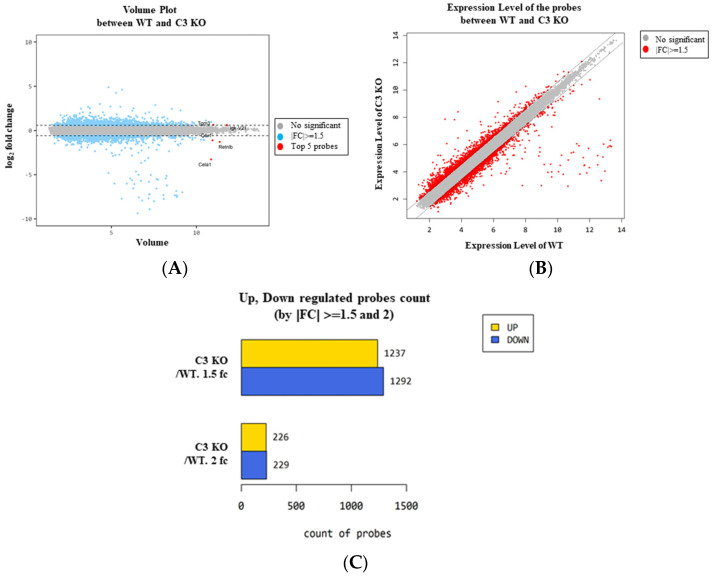
Volume and scatter plots of the normalized microarray in C3 KO mice. (**A**) Volume plot of the WT mice and C3 KO mice. (**B**) Scatter plot of the WT and C3 KO mice. (**C**) Differentially regulated probe count. The number of genes expressed differently between WT and C3 KO mice were represented as 1.5 FC and 2 FC. Abbreviation: WT, wild type; C3 KO, complement C3 knockout; FC, fold change.

To characterize the changes in the global gene expression in response to C3 deficiency-induced constipation, the total RNA extracted from the mid colon tissue of the sixteen-week-old C3 KO mice was hybridized into oligonucleotide microarrays. First, the differential distribution of the global genes between the WT mice and C3 KO mice was confirmed in the normalized data using a volume plot and scatter plot. Genes that upregulated and downregulated more than 1.5-fold were successfully distributed between the two groups, and five genes with high fold changes, including transglutaminase 3, E polypeptide (*TGM3*), carbonic anhydrase 1 (*CAR1*), chymotrypsin-like elastase family, member 1 (*CELA1*), resistin-like beta (*RETNLB*) and immunoglobulin kappa chain variable 21 (*IGK-V21*), were detected in the volume plot (Figure 2A,B). Based on the above comparison, the differentially expressed gene (DEG) (|Fold change (FC)| ≥ 1.5) between the WT and C3 KO mice were detected and these comprised 2529 genes that responded to constipation from the pool of total filtered genes (33,793). Among these, 1237 genes were upregulated, and 1292 genes were downregulated in the C3 KO mice compared to the WT mice. On the other hand, statistical significance was detected in only 545 genes out of the 2529 DEG if cut off value was set to more than 2. These genes exhibited 226 upregulations and 229 downregulations (Table 1, Figure 2C and Supplement Figure S1).

Based on the PANTHER/X ontology analyses, 2529 genes with an FC of more than 1.5 were classified into 20 Gene Ontology (GO) categories after dividing them into upregulated genes and downregulated genes. The upregulated genes included various GO categories, such as immunoglobulin chain (30 genes), response to chemicals (21 genes), molecular transducer activity (16 genes), biological regulation (13 genes), and cell periphery (11 genes). Also, differences in the GO categories including predicted gene (50 genes), immunoglobulin chain (23 genes), response to chemical (22 genes), cell periphery (21 genes), and molecular transducer activity (20 genes) were detected in the downregulated genes (Table 2). Furthermore, each gene was characterized from the lists of total genes upregulated and downregulated in the C3 deficiency-induced constipation in the mid colon of the C3 KO mice. Of the 226 upregulated genes, the largest difference was detected in eukaryotic translation initiation factor 2 subunit 3 and structural gene Y-linked (*Eif2s3y*) genes (29.68-fold), followed by ubiquitously transcribed tetratricopeptide repeat containing, Y-linked (*UTY*, 24.65-fold), lysine demethylase 5D (*KDM5D*, 19.14-fold), immunoglobulin kappa variable 6-32 (*IGKV6-32*, 7.94-fold), olfactory receptor 870 (*Olfr870*, 5.71-fold), and DEAD-box helicase 3 Y-linked (*DDX3Y*, 5.65-fold) (Table 3 and Supplementary Table S1). Also, 229 downregulated genes, including pancreatic lipase (*PNLIP*, -658.36-fold), carboxyl ester lipase (*CEL*, -476.60-fold), carboxypeptidase B1 (*CPB1*, -257.26-fold), chymotrypsin-like (*CTRL*, -214.25-fold), pancreatic lipase-related protein 1 (*PNLIPRP1*, -205.18-fold), and regenerating islet-derived 1 (*REG1*, -187.41-fold) were downregulated in C3 deficiency-induced constipation (Table 4 and Supplementary Table S2). Taken together, these results provide the gene profile in animal models with C3 deficiency-induced constipation.

### 2.3. Reliability of the Expression of Differential Genes Detected in the Microarray Analysis

**Figure 3:**
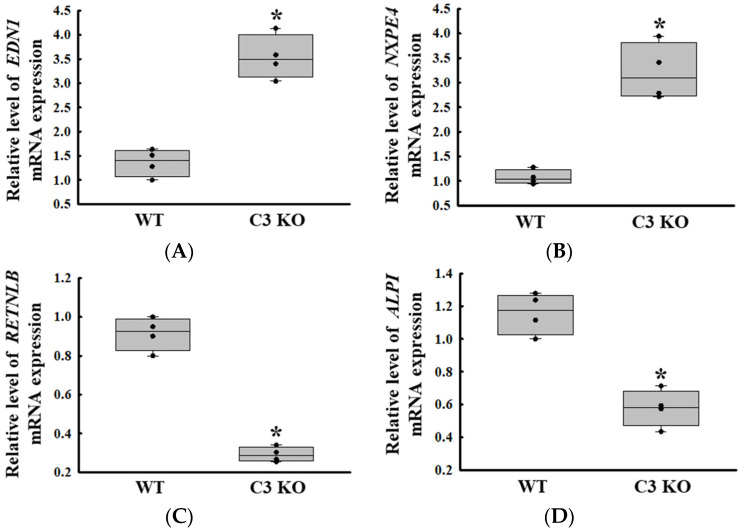
Transcription level of the upregulated genes and downregulated genes. Transcription level of *EDN1* (**A**) and *NXPE4* (**B**). Transcription level of *RETNLB* (**C**) and *ALPI* (**D**). The transcription levels of four genes were measured in the total mRNA of the colon by RT-qPCR using specific primers. The mRNA levels of the four genes were calculated based on the intensity of β-actin as an endogenous control. The preparation of total RNAs was performed on three to five mice per group, RT-qPCR analyses were assayed twice for each total RNA. The data were presented as the mean ± SD. * *p* < 0.05 vs. WT mice. Abbreviation: WT, wild type; C3 KO, complement C3 knockout; *EDN1*, endothelin-1; *NXPE4*, neurexophilin and PC-esterase domain family member 4; *RETNLB*, resistin-like beta; *ALPI*, alkaline phosphatase intestinal; RT-qPCR, quantitative real-time PCR.

We confirmed the reliability of the gene expressions obtained from the microarray analysis. To achieve this, the expression level of two upregulated genes (endothelin-1; *EDN1* and neurexophilin and PC-esterase domain family member 4; *NXPE4*) and two downregulated genes (*RETNLB* and *ALPI*) were measured by RT-qPCR analyses. The expression patterns of these genes were completely reflected in those observed from the microarray analysis of the mid colon in C3 deficiency-induced constipation, although the alteration rate varied. The expression level of the *EDN1* and *NXPE4* genes was increased in the C3 KO mice compared to the WT mice, while those of *RETNLB* and *ALPI* were decreased in the same group (Figure 3).

### 2.4. Biological Functions of Genes Associated with C3 Deficiency-Induced Constipation

**Figure 4:**
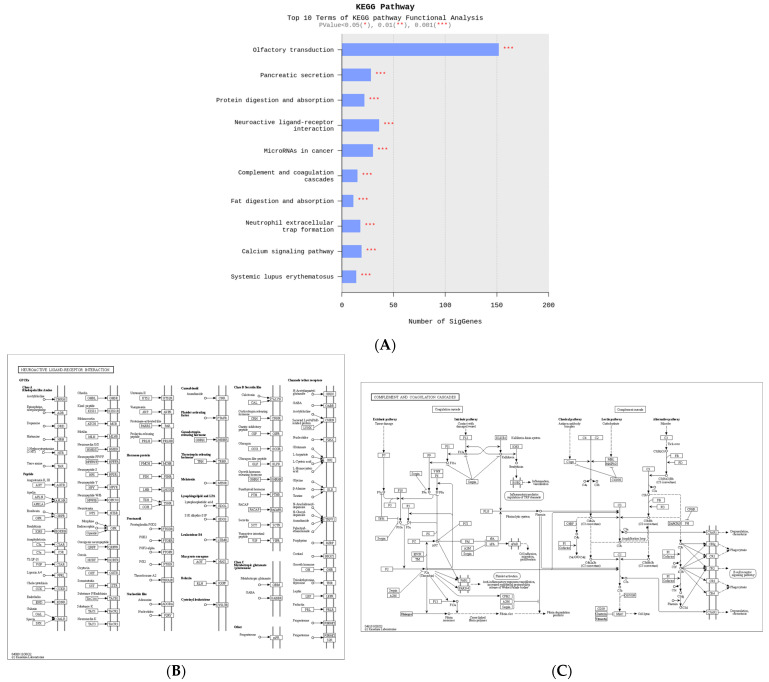
KEGG pathway of functional analyses about differential genes between WT and C3 KO mice. (**A**) Top ten terms with statistical significance in the KEGG pathway. (**B**) Neuroactive ligand–receptor Interaction. (**C**) Complement and coagulation cascades. Abbreviation: WT, wild type; C3 KO, complement C3 knockout; KEGG, Kyoto Encyclopedia of Genes and Genomes.

To identify the biological functions of the genes associated with C3 deficiency-induced constipation, a Kyoto Encyclopedia of Genes and Genomes (KEGG) pathways analysis was conducted on 1237 upregulated genes and 1292 downregulated genes. Among these 2529 genes, the largest number of genes (150 genes) were related to olfactory transduction. Also, these analyses showed that some of these genes were related to ten important pathways, including olfactory transduction, pancreatic secretion, protein digestion and absorption, neuroactive ligand–receptor interaction, microRNAs in cancer, complement and coagulation cascades, fat digestion and absorption, neutrophil extracellular trap formation, calcium signaling pathway and systemic lupus erythematosus (Figure 4A). Specifically, several DEGs such as vitronectin (*VTN*), *C3*, *C4A*, CD59b antigen (*CD59b*), integrin alpha X (*ITGAX*), fibrinogen alpha chain (*FGA*), coagulation factor III (*F3*), CD55 molecule, decay accelerating factor for complement (*CD55*), kininogen 1 (*KNG1*), complement C3d receptor 2 (*CR2*), *C7*, fibrinogen gamma chain (*FGG*) and peptidase inhibitor, clade A, member 1C (*SERPINAL1C*) belonged to the neuroactive ligand–receptor interaction pathway (Figure 4B). In addition, the complement and coagulation cascades pathway included several marker genes including *2210010C04Rik*, *Protease*, serine 2 (*PRSS2*), amylase alpha 2B (*AMY2B*), *PRSS1*, trypsin (TRY)4, cholecystokinin A receptor (*CCKAR*), *TRY5*, *TRY10*, gamma-aminobutyric acid A receptor, subunit alpha 4 (*GABRA4*), glucagon-like peptide 1 receptor (*GLP1R*), *1810009J06Rik*, *C3*, adrenergic receptor, alpha 1d (*ADRALD*), *PRSS3*, trace amine-associated receptor 7D (*TAAR7D*) and *9230113P08Rik* for GI-related diseases (Figure 4C). Therefore, these results suggest that the DEGs associated with C3 deficiency-induced constipation are closely associated with olfactory transduction, neuroactive ligand–receptor interaction, microRNAs in cancer, pancreatic secretion, protein digestion and absorption, and the calcium signaling pathway.

### 2.5. Selection of the ALPI Gene as a New Treatment Target for C3 Deficiency-Induced Constipation and Verification of Its Function in Acetate-Treated Epithelial Cells

**Figure 5:**
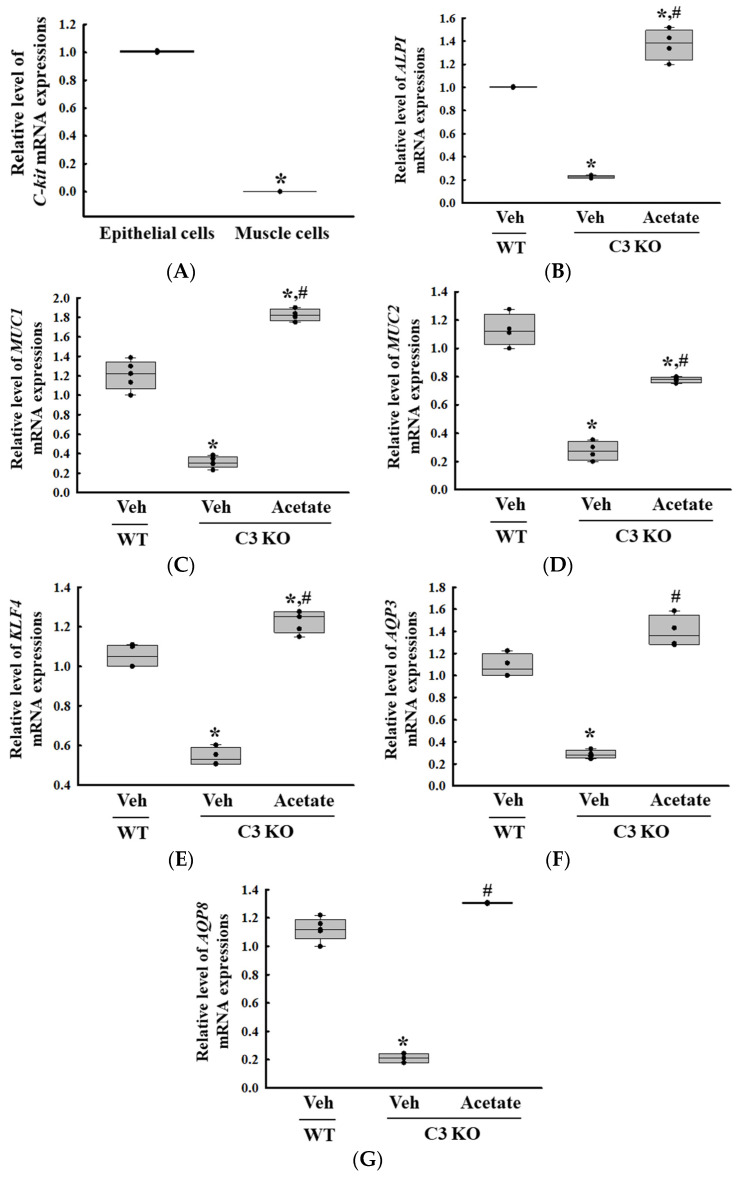
Transcription level of *C-kit, ALPI* and mucin secretion genes in epithelial cells from intestine of C3 KO mice after acetate treatment. (**A**) Transcription level of *C-kit* gene in isolated epithelial cells and muscle cells. * *p* < 0.05 vs. Muscle cells. (**B**) Transcription level of the *ALPI* gene in acetate-treated C3 KO epithelial cells. (**C**–**G**) Transcription level of *MUC1*, *MUC2*, *KLF4*, *AQP3* and *AQP8* in acetate-treated C3 KO epithelial cells. The transcription levels of each gene were measured in the total mRNA of the epithelial cells by RT-qPCR using specific primers. The mRNA levels of each gene were calculated based on the intensity of β-actin as an endogenous control. The preparation of total RNAs was performed on three to five wells per group, RT-qPCR analyses were assayed twice for each total RNA. The data were presented as the mean ± SD. * *p* < 0.05 vs. no treated group. # *p* < 0.05 vs. vehicle-treated C3 KO group. Abbreviation: WT, wild type; C3 KO, complement C3 knockout; *MUC*, mucin; *AQP*, aquaporin; *ALPI*, alkaline phosphatase intestinal; C-kit, receptor protein kinase; PBS, phosphate buffer saline; RT-qPCR; quantitative real-time PCR.

Next, we tried to identify the novel target gene associated with C3 deficiency-induced constipation based on the changes in the global gene expression in the colon. Among all the altered expression of gene, the *ALPI* gene was selected as the novel functional gene for constipation because the expression level of this gene was downregulated in Lop-induced constipation and C3 deficiency-induced constipation as well as the fact that the deficiency of the protein encoded by *ALPI* had been investigated as the cause of inflammatory bowel disease (IBD) [10,13]. Thus, the expression of the *ALPI* gene and its effects on mucin secretion ability and water retention capacity in C3 deficiency-induced constipation was first investigated in the epithelial cells derived from the C3 KO mice. Hence, alterations in the expression levels of the mucin (*MUC*) and aquaporins (*AQP*) genes were analyzed in C3-deficient epithelial cells after acetate treatment because in an earlier study 1 mM acetate treatment successfully induced the expression of the *ALPI* gene in pluripotent stem cell derived intestinal epithelial cell layers [14]. Briefly, the successful isolation of epithelial cells from the colon of C3 KO mice was verified by the detection of a high level of receptor protein kinase (*C-kit*) mRNA compared to that of muscle cells (Figure 5A). The expression level of the *ALPI* gene was lower in the epithelial cells derived from the C3 KO mice than in those from the WT mice. However, these levels were remarkably enhanced to levels above those of the WT mice cells after the acetate treatment (Figure 5B). These results show that the acetate-treated epithelial cells from C3 KO mice were suitable for analyzing the recovery effects of the *ALPI* gene during C3 deficiency-induced constipation. Furthermore, the expression levels of mucin, cell surface-associated (*MUC1*), oligomeric mucus/gel-forming (*MUC2*), Kruppel-like factor 4 (*KLF4*), *AQP3*, and *AQP8* genes were significantly increased in the epithelial cells during the upregulation of the acetate-induced *ALPI* gene (Figure 5C). Therefore, all the results from the study in epithelial cells suggest that the upregulation of *ALPI* expression may be tightly linked to the improvement of C3 deficiency-induced constipation.

### 2.6. Verification of the ALPI Gene as a New Treatment Target for C3 Deficiency-Induced Constipation in Acetate-Treated C3 KO Mice

**Figure 6:**
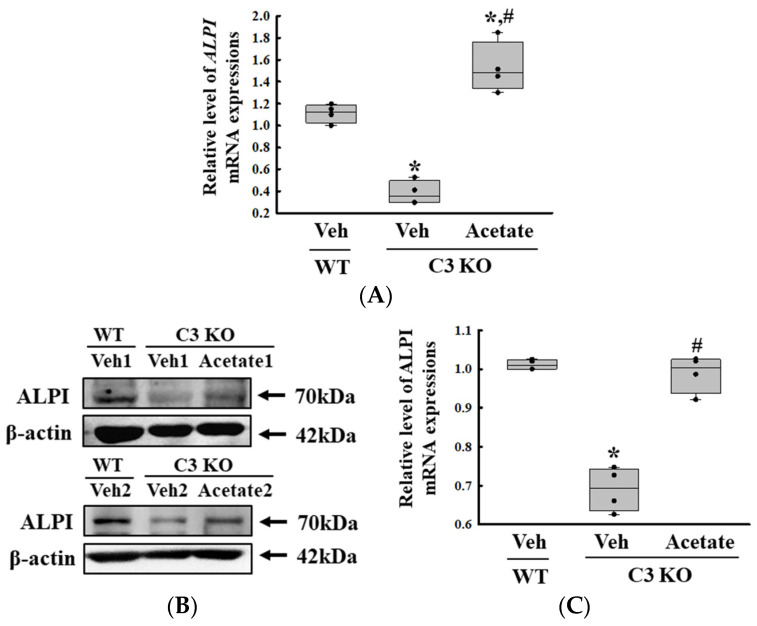
Transcription and expression levels of the *ALPI* gene and proteins. (**A**) Transcription level of the *ALPI* gene in acetate-treated C3 KO mice. The transcription levels of the *ALPI* gene were measured in the total mRNA of the epithelial cells by RT-qPCR using specific primers. The mRNA levels of each gene were calculated based on the intensity of β-actin as an endogenous control. The preparation of total RNAs was performed on three to five mice per group, RT-qPCR analyses were assayed twice for each total RNA. (**B**) Expression levels of ALPI proteins in acetate-treated C3 KO mice. After collecting the mid colons from the C3 KO mice, the expression levels of ALPI proteins were assessed in tissue homogenates using the specific primary antibody and densitometry. The tissue homogenates were prepared from four to six mice per group and the Western blot was analyzed twice for each sample. After normalizing with β-actin as endogenous control, the relative level of ALPI expression was presented as a relative value to the vehicle-treated WT group (**C**). The data were presented as the mean ± SD. * *p* < 0.05 vs. no treated group. # *p* < 0.05 vs. vehicle-treated C3 KO group. Abbreviation: WT, wild type; C3 KO, complement C3 knockout; *ALPI*, alkaline phosphatase intestinal; RT-qPCR, quantitative real-time PCR.

**Figure 7:**
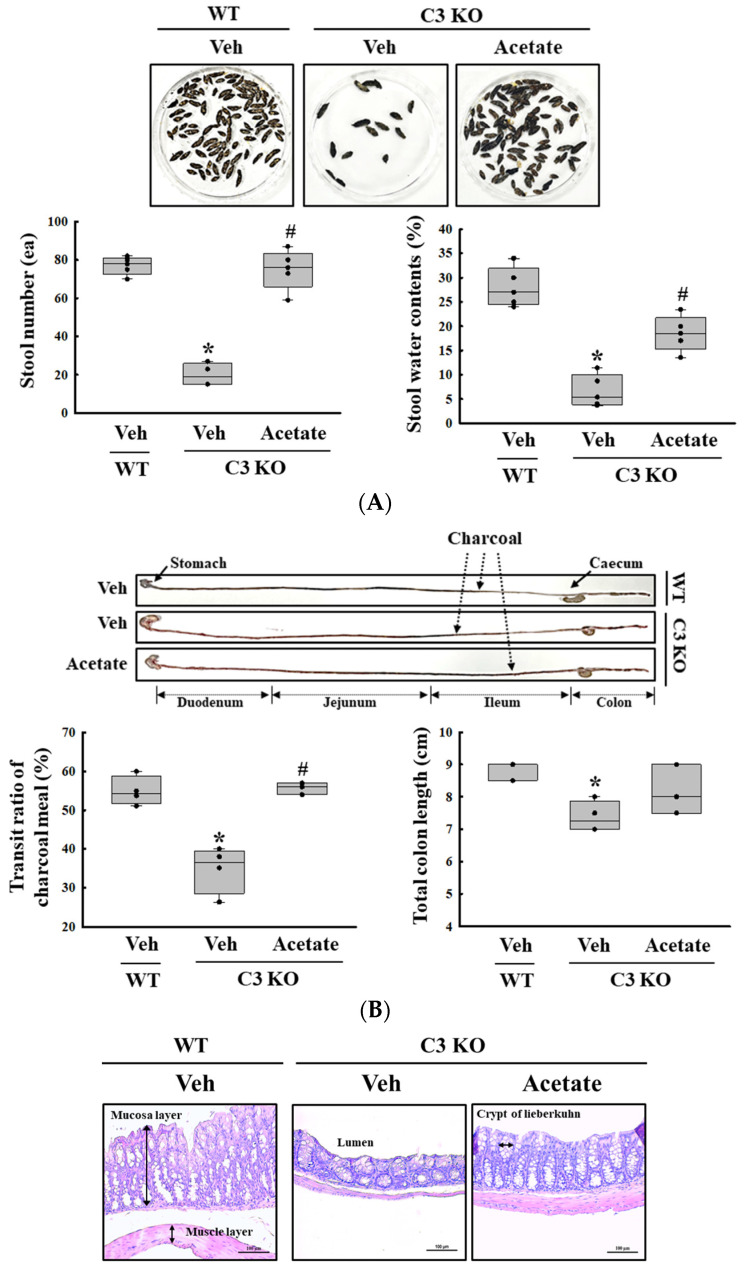

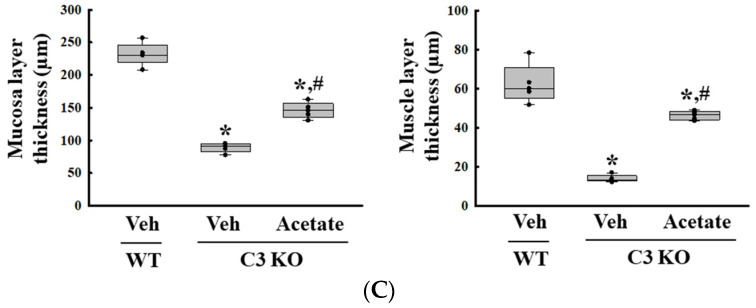
Stool parameters, GI transit and histological structure of colon in acetate-treated C3 KO mice. (**A**) Stool parameters. The preparation of the total stools was performed on three to five mice per group and each parameter was analyzed twice for each sample. (**B**) GI transit and total length of GI tract. After collection from the stomach to the anus, the entire GI tract was arranged in a row, and the location of the charcoal meal was marked with arrows. Transit ratio of charcoal meal and total length of GI tract were measured as described in materials and method. The preparation of the GI tract was performed on three to five mice per group, and each parameter was analyzed twice for each mouse. (**C**) Histological structure of mid colon. The H&E-stained tissue sections were prepared from tissue samples from three to five mice per group, and the pathological factors were analyzed twice for each stained tissue. The data are presented as the mean ± SD. * *p* < 0.05 vs. no treated group. # *p* < 0.05 vs. vehicle-treated C3 KO group. Abbreviation: WT, wild type; C3 KO, complement C3 knockout; *ALPI*, alkaline phosphatase intestinal; H&E, hematoxylin and eosin.

**Figure 8:**
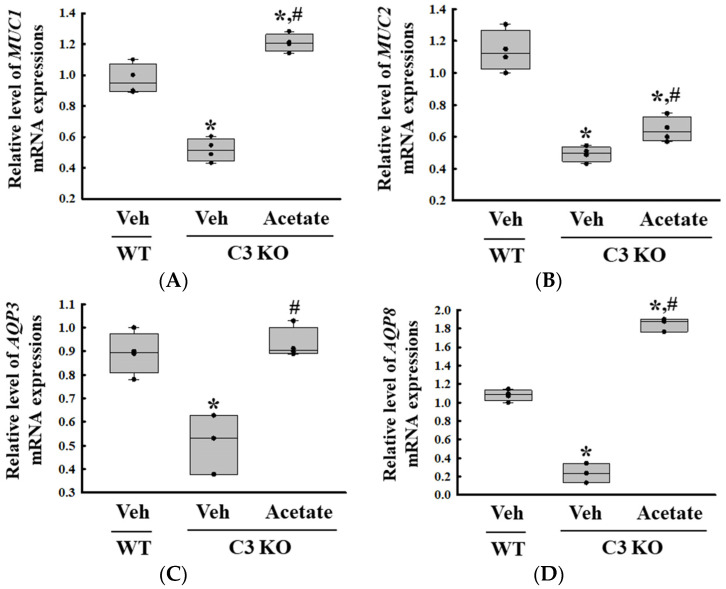
Transcription level of (**A**) *MUC1*, (**B**) *MUC2*, (**C**) *AQP3* and (**D**) *AQP8* gene in the colon tissue of acetate-treated C3 KO mice. The transcription levels of four genes were measured in the total mRNA of the colon tissues by RT-qPCR using specific primers. The mRNA levels of each gene were calculated based on the intensity of β-actin as an endogenous control. The preparation of total RNAs was performed on three to five mice per group, RT-qPCR analyses were assayed twice for each total RNA. The data were presented as the mean ± SD. * *p* < 0.05 vs. no treated group. # *p* < 0.05 vs. vehicle-treated group. Abbreviation: WT, wild type; C3 KO, complement C3 knockout; MUC, mucin; AQP, aquaporin.

Finally, we verified whether the improvement of C3 deficiency-induced constipation due to *ALPI* upregulation in acetate-treated epithelial cells could be reproduced in the C3 KO mice. To achieve this, alterations in the excretion parameters, GI motility, histological structure of the mid colon, mucin secretion ability, and water retention capacity were analyzed in the C3 KO mice treated with 50 mg/kg acetate for 24 h. First, the transcription levels of the *ALPI* gene and the expression level of ALPI proteins were upregulated in the mid colon of the acetate-treated C3 KO mice when compared with the vehicle-treated C3 KO mice (Figure 6A,B). Also, the decrease in the number and water content of the stools in the vehicle-treated C3 KO mice significantly recovered after acetate treatment, although the recovery rate was not completed (Figure 7A). Furthermore, similar recovery patterns in acetate-treated mice were detected in GI motility, colon length, and the histological structure of the colon. The transit ratio of the charcoal meal and the colon length were lower in the vehicle-treated C3 KO mice than in the untreated WT mice. However, these levels were remarkably increased in the acetate-treated C3 KO mice compared to the vehicle-treated C3 KO mice (Figure 7B). The acetate treatment in C3 KO mice with constipation phenotypes induced an increase in the thickness of the mucosa and the muscle layer although these did not fully recover to normal levels (Figure 7C). Moreover, the markers for mucin secretion ability and water retention capacity showed similar alteration patterns after acetate treatment. The decrease in *MUC1*, *MUC2*, *AQP3*, and *AQP8* expression was seen in C3 KO mice recovered after the acetate treatment (Figure 8A–D). Taken together, the results of the above study suggest that the upregulation of *ALPI* expression could contribute to the improvement of C3 deficiency-induced constipation in C3 KO mice. In addition, these results suggest that alterations in the *ALPI* gene expression can act as one of the novel causes of C3 deficiency-induced constipation.

## 3. Discussion

C3 deficiency-induced constipation is being actively studied in the context of inflammatory response, regulatory mechanism of ENS, gut microbiota composition, and application of therapeutic drugs [7,8,9,15]. However, many additional studies are needed to understand the pathophysiological mechanisms associated with constipation due to C3 deficiency and the therapeutic targets for its management in animal models. This study aimed to supplement the current insufficient scientific evidence available on the subject. We sought to identify new genes and their functions associated with C3 deficiency-induced constipation. In this study, the alterations in the global gene expression were characterized in the mid colon of sixteen-week-old C3 KO mice with the constipation phenotype. Among the genes characterized, the *ALPI* gene was selected, and its function was analyzed in primary epithelial cells and C3 KO mice. Our results provide novel scientific information on the gene profiles associated with C3 deficiency-induced constipation, even though various genes whose functions remain unidentified have also been included in this list. In addition, these results suggest for the first time that the *ALPI* gene has an important function in the pathogenesis of constipation, although further clinical studies are needed to establish this.

Even though various studies are being conducted on the treatment of constipation of varied etiology, the alterations in the global gene expression in response to the constipation phenotypes have been characterized in only two studies in animals and patients. Approximately 797 genes (581 upregulated and 216 downregulated) were differentially expressed in the mid colon of Lop-induced constipation in rats. Among them, the major genes in the downregulated category included *SLC9A5*, *KLK10*, *FGF15*, and *ALPI*, whereas the major genes in the regulated category were cytochrome P450 (CYP) 2B2 (*CYP2B2*), *ACE*, *G6PC*, and *SETBP1* [10]. Also, a similar characterization of the global gene expression was analyzed in IBS patients with constipation (IBS-C). Approximately 1270 genes (False discovery rate (FDR) < 0.05) were detected as DEGs between IBS-C vs. age-/sex-matched healthy controls (HCs). Five genes including aldehyde dehydrogenase 9, family member A1 (*ALDH9A1*), claudin 15 (*CLDN15*), enhancer of zeste 2 polycomb repressive complex 2 subunit (*EXH2*), ring finger protein 19A, RBR E3 ubiquitin protein ligase (*RNF19A*), and YIP1 interacting factor homolog B, membrane trafficking protein (*YIF1B*) showed significant positive correlations between the microarray and nCounter^®^ values [12]. In the current study, the global gene expression in the mid colon was compared between C3 KO mice with constipation phenotypes and WT mice. A total of 2529 genes differentially expressed between the WT and C3 KO mice were identified from the mid colon and these comprised 1237 upregulated genes and 1292 downregulated genes. The major genes in the downregulated category included *Eif2s3y*, *UTY*, *KDM5D*, *IGKV6-32*, *Olfr870*, *DDX3Y*, *EDN1*, and *NXPE4*, whereas the major genes in the upregulated category were *PNLIP*, *CEL*, *CPB1*, *CTRL*, *PNLIPRP1*, *REG1*, *RETNLB* and *ALPI*. When compared to earlier studies, the number of altered genes with differential expressions was higher in our study than in the previous two studies. In lop-induced constipation, the total number of the upregulated genes was 2.7 times higher than the downregulated genes, but a similar number of upregulated and downregulated genes was observed between mice with WT and C3 deficiency-induced constipation [10]. Also, approximately 25 genes were found to be altered in all three types of constipation including IBS-C, Lop-induced constipation, and C3 deficiency-induced constipation. Of these, ten genes including annexin A8 (*ANXA8*), MIS18 kinetochore protein A (*MIS18A*), CDC42 effector protein 5 (*CDC42EP5*) and ankyrin repeat domain 37 (*ANKRD37*) showed the same upregulation pattern in all the three models [10,12].

Meanwhile, we selected seven genes that were expected to play an important role in C3 deficiency-induced constipation from a total of 2529 genes differentially expressed between WT and C3 KO mice. Among them, the five downregulated genes, including *PRSS2*, trefoil factor 2 (*TFF2*), *RETNLB*, solute carrier family 9 (sodium/hydrogen exchanger) (*SLC9A5*), member 3 (*SLC9A3*), and *ALPI*, and the two upregulated genes including *EDN1* and *NXPE4* were thought to be candidates associated with C3 deficiency-induced constipation. Scientific evidence on the role of these genes in constipation has been reported in several earlier studies. First, in the present study, the *PRSS2* gene encoded trypsinogen-2 (anionic trypsin) was downregulated 94.18-fold in C3 deficiency-induced constipation. This protein was preferentially secreted from the pancreas and cleaved into an active form in the small intestine. In IBS patients, the enhanced level of this protein was associated with an increase in intestinal permeability and an induction of intestinal hypersensitivity [16]. Also, the proteins encoded by the *TFF2* gene produced from gastrointestinal mucosa stimulate immune regulation and recovery, affect the healing of the mucosal layer, and suppress the inflammatory response in the rectum [17]. In our study, the expression of this gene was decreased 10.82-fold in the C3 KO mice compared to the WT mice. The *RETNLB* gene encodes for the resistin-like beta protein, which is associated with mucin secretion, although C3 deficiency-induced constipation decreases their transcription -2.43-fold [18]. The *SLC9A3* gene is considered to be a Na/H exchanger in the epithelial brush border, and defects in this gene can induce diarrhea [19]. The decrease was -2.11-fold for both genes *RETNLB* and *SLC9A3*. Furthermore, the *ALPI* gene was downregulated 2.06-fold in the mid colon during C3 deficiency-induced constipation. This gene plays an important role in the homeostasis of the intestine, and a defect in this gene induces inflammatory responses and an imbalance of intestinal microbiota [10]. Moreover, two upregulated genes associated with C3 deficiency-induced constipation were also identified. The *EDN1* gene encodes the endothelin 1 protein, which is associated with pulmonary hypertension, lipid metabolism, and insulin resistance [20,21,22]. It regulates the motility of the GI and intestinal passage time. The symptoms of constipation improved by the suppression of secretion of this protein [23]. *NXPE4* is a cytoplasmic protein expressed in the salivary gland, colon, and rectum [24]. In the present study, the expression levels of the *EDN1* and *NXPE4* genes were upregulated 2.06- and 2.00-fold, respectively, during C3 deficiency-induced constipation.

In this study, we identified the *ALPI* gene as a constipation-associated novel gene because it was commonly detected in the Lop-induced constipation model and the C3 deficiency-induced constipation model of the present study [10]. The *ALPI* gene encodes intestinal alkaline phosphatase, an endogenous protein expressed in the intestinal epithelium to maintain gut homeostasis [25]. It is released from the enterocyte apical membrane into the lumen by lysophosphatidylcholine and is then incorporated into intracellular lipid droplets and surfactant-like particles [26,27]. The alkaline phosphatase plays a pivotal role in energy regulation and intestinal barrier functions such as the absorption of long-chain fatty acids, regulation of duodenal bicarbonate secretion, detoxification of bacterial endotoxin, and control of the transmucosal passage of bacteria [25,28]. Based on the physiological role of these proteins, some studies have reported the exogenous administration of these proteins as supplements to treat animals and humans to relieve intestinal and systemic inflammation in various diseases [29]. The supplementation decreases the levels of C-reactive proteins and reduces inflammation in ulcerative colitis and dextran sodium sulfate (DSS)-induced colitis [30,31]. A defect in the gene leading to a deficiency in the protein in mice induces an increase in the colitis score in DSS-induced models and the bacterial translocation caused by intestinal ischemia [31,32]. Therefore, the results of the present study provide the first evidence for the novel function of the *ALPI* gene in constipation implying that the suppression of *ALPI* expression could be tightly linked to the induction of constipation. However, many additional studies on the mechanism are needed.

Furthermore, to confirm our findings, we examined whether the recovery of *ALPI* expression can improve the constipation caused by C3 deficiency. The upregulation of *ALPI* expression was induced by butyrate and acetate treatments as an earlier study indicated that the transcription of the *ALPI* gene was increased in induced pluripotent stem cells (iPSC)-derived human intestinal epithelial cells treated with 1 mM acetate [14]. Subsequently, the epithelial cells wherein *ALPI* gene expression levels were restored were analyzed for the mucin secretion ability and water retention capacity with respect to the functions of the epithelial cells in the colon [33]. In this study, the upregulation of *ALPI* expression by the acetate treatment was accompanied by the upregulation of *MUC1*, *MUC2*, *AQP3*, and *AQP8* transcripts in C3-deficient epithelial cells and C3 KO mice.

## 4. Materials and Methods

### 4.1. Animal Care and Use

Firstly, the animal protocols used in this study were reviewed in terms of science, ethics and safety, and approved by the Pusan National University-Institutional Animal Care and Use Committee (PNU-IACUC; Approval Number PNU-2023-0407). All mice were provided from Laboratory Animals Banks in the Korea Food and Drug Administration (KFDA). They were provided ad libitum access to tap water and a standard irradiated chow diet (Samtako BioKorea Co., Osan, Republic of Korea). Also, these mice were bred at the PNU-Laboratory Animal Resources Center (LARC), which is accredited by the KFDA (Accredited Unit Number: 000231) and the Association for Assessment and Accreditation of Laboratory Animal Care (AAALAC, Accredited Unit Number: 001525). The LARC provided an international standard environmental condition for animal breeding including 23 ± 2 °C temperature, 50 ± 10% relative humidity, 12 h of light/dark cycle (lights on at 08:00 h and off at 20:00 h) and a specific pathogen free (SPF) state. The WT and C3 KO mice were used for microarray analyses, acetate treatment, and constipation phenotypes analyses.

### 4.2. Experimental Designs of C3 KO Mice for Microarray Analyses

First of all, the WT and C3 KO mice that need for microarray analyses were produced by crossbreeding between heterogeneous type (HT) mice of C3 gene, as described in our previous studies [6,7]. When WT and C3 KO mice grew up to the sixteen-week-old, these mice were divided into WT mice (n = 7) and C3 KO mice (n = 7). The stool parameters were analyzed using stools collected from a metabolic cage (Daejong Instrument Industry Co., Ltd., Seoul, Republic of Korea) after 1 day of breeding. A charcoal meal was administrated into each mouse for GI transit analyses as described in previous study [6]. Finally, all the mice were euthanized based on the American Veterinary Medical Association (AVMA) Guidelines using CO_2_ gas with a minimum purity of 99.0%. To achieve this, a cage containing dirty bedding and mice was placed in the gas chamber for mice and CO_2_ gas of 99.0% purity was injected into this chamber without pre-charging, with a fill rate of ~50% of the chamber volume per minute. After then, their final death was confirmed by ascertaining cardiac and respiratory arrest, or dilated pupils and fixed body. The GI tracts were collected from the mice of the subset group for GI transit, and the mid colon tissues were collected for histopathological and microarray analyses because this region plays an important role in the process of reabsorption of water and compaction of the digested food mixtures into stools [34].

### 4.3. Experimental Designs for the Acetate Treatment of C3 KO Mice

**Figure 9:**
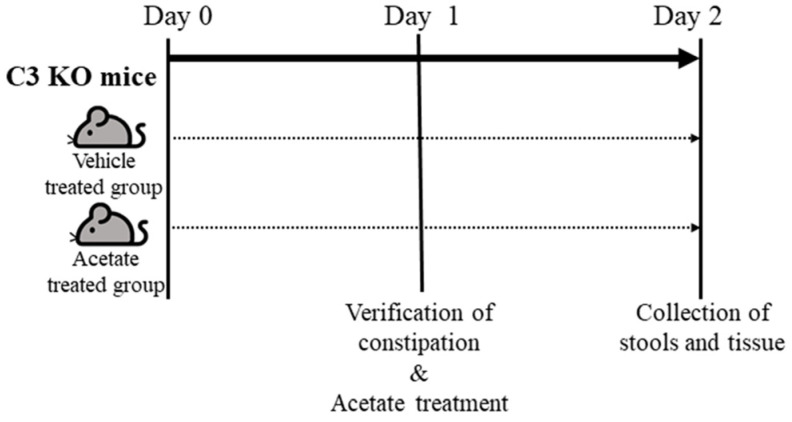
Schedule for acetate treatment into C3 KO mice. After allocation of the WT and C3 KO mice into one of three experimental groups, mice of each group were treated with 5 mM acetate or 1× PBS, and subsequentially collected the stools and colon tissue from these mice. Abbreviation: WT, wild type; C3 KO, complement C3 knockout; RT-qPCR, quantitative real-time PCR; PBS, phosphate buffer saline.

Acetate treatment was used to upregulate the expression of *ALPI*, as described in our previous studies (Figure 9) [14]. Sixteen-week-old WT (n = 7) and C3 KO mice (n = 14) were divided into groups as follows; Untreated WT mice (n = 7), vehicle-treated C3 KO mice (n = 7) and acetate-treated C3 KO mice (n = 7). The upregulation of *ALPI* expression was induced by the oral gavage of a single dose of acetate (50 mg/kg, Sigma-Aldrich Co., St. Louis, MO, USA) in 1× phosphate buffer saline (PBS) solution for a day, while the mice in the vehicle-treated group were injected with the same volume of 1× PBS solution. At 24 h after the final acetate treatment, all mice were individually held in metabolic cages (Daejong Instrument Industry Co., Ltd.) for two days to analyze the GI motility and excretion parameters and were euthanized using CO_2_ gas. Also, the colon tissue samples were collected and stored at −80 °C until mRNA and protein analyses could be carried out.

### 4.4. Analyses of the Stool Parameters

All mice were individually held in metabolic cages (Daejong Instrument Industry Co., Ltd.) throughout the experiment to obtain uncontaminated stool samples, as described in a previous study [7,8]. Briefly, the stools excreted from each mouse were collected at 9 a.m. for two days, weighed twice using an electronic balance, and their number was counted twice. Also, their morphology was analyzed after taking images with a digital camera. The stool water content was also calculated as follows:

Stool water content = (A − B)/A × 100

where A is the weight of fresh stools collected from mice of the subset groups, while B is the weight of stools after drying at 60 °C for 12 h.

### 4.5. GI Motility Analysis

The charcoal transit ratio was used to determine GI motility as described in previous studies with some modifications [35]. Firstly, the WT and C3 KO mice fasted for 18 h except for water ad libitum to remove food mixture from the GI tracts. They were administrated 0.5 mL of charcoal meal (3% suspension of activated charcoal in 0.5% aqueous methylcellulose) (Sigma-Aldrich Co.). After 30 min of treatment, all mice were euthanized using CO_2_ after, and total GI tract was collected from the abdominal cavity. The charcoal transit ratio in each mouse was calculated as follows:

Charcoal transit ratio (%) = [(Total intestine length − Transit distance of charcoal meal)/ Total intestine length] × 100

### 4.6. Histopathological Analysis

After collecting the mid colons from the WT and C3 KO mice, these tissues were fixed in 10% formalin for 48 h, and embedded in paraffin wax. These tissues were sectioned into 4 μm thick slices using Microtome (Leica Microsystems, Heerbrugg, Switzerland), and then stained with hematoxylin & eosin (H&E; Sigma-Aldrich Co.) to distinguish between the nuclear and cytoplasmic parts. An alteration on the histological structure of these stained colon sections were observed under a light microscope, after which the thickness of the mucosal layer and muscle layer in the mid colon were observed using the Leica Application Suite (Leica Microsystems).

### 4.7. Quantitative Real-Time PCR (RT-qPCR) Analysis

The relative quantities of the mRNAs for gene identified from microarray were confirmed using RT-qPCR analysis. Briefly, the total RNAs were purified from frozen mid colon tissues using RNAzol (Tet-Test Inc., Friendswood, TX, USA) as manufacture’s protocol. Their concentration was determined using ND-2000 Spectrophotometer (NanoDrop, Wilmington, DE, USA), 4 μg of total RNAs were used to synthesis complementary DNA (cDNA) under the specific conditions including oligo-deoxythymidine (dT) primer (Thermo Fisher Scientific Inc., Waltham, MA, USA), 2′-deoxynuclease 5′-triphosphate (dNTP) and reverse transcriptase (Superscript II, Thermo Fisher Scientific Inc., #18064-014). After then, each gene was amplified using a cDNA template, 2× Power SYBR Green (TOYOBO Co., Osaka, Japan), and specific primer (Supplementary Table S3) as described in a previous study [36]. The reaction cycle at which PCR products exceeded the fluorescence intensity threshold during the exponential phase of PCR amplification was considered the threshold cycle.

### 4.8. Western Blot Analysis

After collecting the mid colon (40 mg) from each mouse, tissue homogenate was prepared in Pro-prep Protein Extraction Solution (iNtRON Biotechnology Inc., Seongnam, Republic of Korea) using Polytron PT-MR 3100D Homogenizer (Kinematica AG, Lusern, Switzerland). The protein concentration of each sample was determined using Bicinchoninic acid Protein Assay (BCA) Kit (Thermo Fisher Scientific Inc.), and then 30 µg of protein were used to separate each protein on the 10% dodecyl sulfate-polyacrylamide gel electrophoresis (SDS-PAGE). After electrophoresis for 1.5 h, these separated proteins were transferred onto nitrocellulose membranes at 40 V for 2 h. Each membrane was incubated in 4 °C with different primary antibody follows: ALPI (Invitrogen Co., Ltd., Carlsbad, CA, USA, #PA5-22210), β-actin (Cell Signaling Technology Inc., Cambridge, MA, USA, #4967). After removing non-specific antibodies using washing solution (137 mM NaCl, 2.7 mM KCl, 10 mM Na_2_HPO_4_ and 0.05% Tween 20), and this membrane was sequentially incubated with horseradish peroxidase (HRP)—conjugated goat anti-rabbit IgG (TransGen Biotech Co., Ltd., Beijing, China, G21234) for 1 h. Finally, chemiluminescent substrates (Dogen, Seoul, Republic of Korea, DG-WF 100) were reacted with HRPs on the immunoblots, and the intensity of each band on the immunoblot was measured using FluorChem^®^ FC2 Imaging system (Alpha Innotech Corporation, San Leandro, CA, USA).

### 4.9. Microarray Analysis

An alteration on the gene profiles for C3 deficiency-induced constipation were analyzed using microarray analysis as described in previous studies [37]. Briefly, total RNAs were purified according to methods previously applied to the RT-qPCR analysis, and their purity were analyzed Agilent 2100 Bioanalyzer (Agilent Technologies, Palo Alto, CA, USA). Also, the Affymetrix Whole Transcript Expression array process was performed using the GeneChip^TM^ Whole Transcript PLUS Reagent Kit (ThermoFisher Scientifc Inc.). Total cDNA for mRNA was synthesized using the GeneChip^TM^ Whole Transcript Amplification Kit (ThermoFisher Scientifc Inc.), and biotin-labeled with terminal deoxynucleotidyl transferase (TdT) using the GeneChip^TM^ HT WT Terminal Labeling Kit (ThermoFisher Scientific Inc.). After then, these labeled DNA targets (5.5 μg) were hybridized to the Affymetrix GeneChip^TM^ Mouse 2.0 ST Array (ThermoFisher Scientific Inc.) at 45 °C for 16 h. The unbound fragments were washout from arrays, and the specific targets were stained on a GeneChip^TM^ Fluidics Station 450 (ThermoFisher Scientific Inc.). Finally, the target signals were scanned on a GCS3000 Scanner (Affymetrix, Santa Clara, CA, USA) and their values were calculated using the Affymetrix^®^ GeneChip™ Command Console^®^ Software (AGCC).

After normalizing using a robust multi-average (RMA) method implemented in Affymetrix^®^ Power Tools (APT, Affymetrix), the result with gene level RMA analysis was exported, and DEG analysis was performed. The statistical significance for transcription level of each gene was determined using an independent Tukey’s post hoc *t*-test and the fold change in which the null hypothesis was that no difference exists among groups. The FDR was controlled by adjusting the *p*-value using the Benjamini–Hochberg algorithm. In case of the DEG set, hierarchical cluster analysis was performed using complete linkage and Euclidean distance as a similarity measure. Gene-enrichment and functional annotation analysis for a significant probe list was performed using Gene Ontology (GO, http://geneontology.org) and Kyoto Encyclopedia of Genes and Genomes (KEGG, http://kegg.jp). All data analysis and visualization of differentially expressed genes were conducted using R 3.3.2 (www.r-project.org).

### 4.10. Acetate Treatment of Epithelial Cells Derived from the Colon of C3 KO Mice

**Figure 10:**
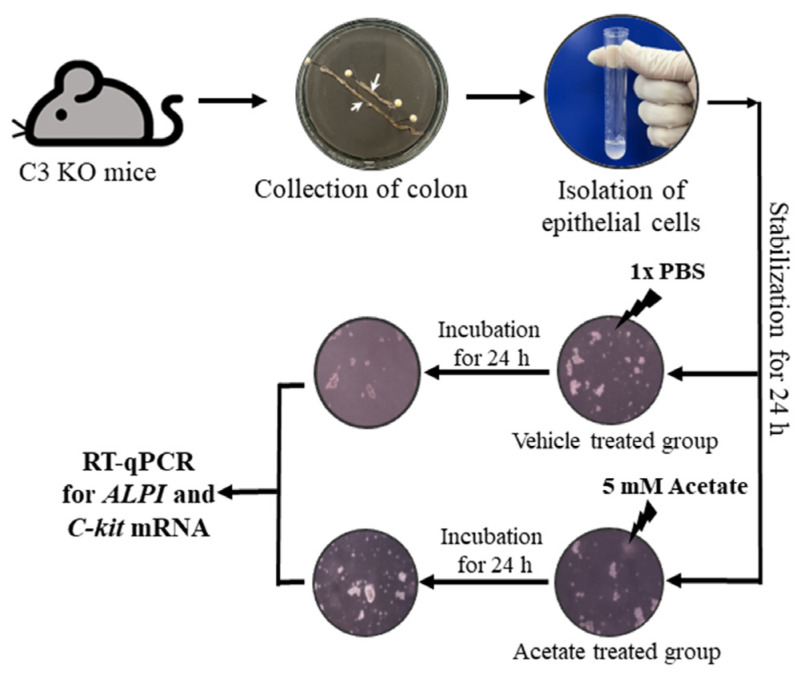
Scheme for purification of epithelial cells from intestine of C3 KO mice and acetate treatment. After collection of the epithelial cells from the intestines of WT and C3 KO mice, these cells were treated with 5 mM acetate or 1× PBS, and the expression of several genes were evaluated by RT-qPCR analysis using specific primers. Abbreviation: WT, wild type; C3 KO, complement C3 knockout; *ALPI*, alkaline phosphatase intestinal; C-kit, receptor protein kinase; PBS, phosphate buffer saline; RT-qPCR; quantitative real-time PCR.

The epithelial cells for the acetate treatment were collected from the colon of the WT and C3 KO mice, as described in previous studies with some modifications [38]. Briefly, the colons of the mice were extracted, and the impurities were eliminated in a Ca^2+^ free Hank’s solution (Gibco, Grand Island, NE, USA). Subsequently, the muscular layer of the intestines was excised. The cells were then dispersed using enzyme solution (albumin (MP Biomedicals, Irvine, CA, USA), trypsin inhibitors (Sigma-Aldrich Co.), and collagenase type 2 (Worthington Biochemical Co., Lakewood, NJ, USA)). For the acetate treatment, the epithelial cells were seeded at a density of 1 × 10^7^ cells/10 mL in a 25 cm^2^ surface area culture flask and grown to 70-80% confluence in a 37 °C incubator. After that, they were incubated with 5 mM acetate for another 24 h (Figure 10). The treatment concentrations of acetate to upregulate *ALPI* expression were based on a previous study [14]. Next, the cells harvested from the 25 cm^2^ culture flask were used for total RNA isolation and RT-qPCR analysis.

### 4.11. Statistical Analysis

Statistical significance was evaluated using one-way analysis of variance (ANOVA) (SPSS for Windows, Release 10.10, Standard Version, Chicago, IL, USA) followed by Tukey’s post hoc *t*-test for multiple comparisons. Data are presented as the mean ± SD (standard deviation). *p* < 0.05 was considered to indicate a statistically significant difference.

## 5. Conclusions

In this study, we sought to identify a novel gene associated with C3 deficiency-induced constipation using microarray analyses. The present results suggest that C3 deficiency-induced constipation may be associated with 1237 upregulated genes including *PNLIP*, *CEL*, *CPB1*, *CTRL*, *PNLIPRP1*, and *REG1* as well as 1292 downregulated genes including *Eif2s3y*, *UTY*, *KDM5D*, *IGKV6-32*, *Olfr870*, and *DDX3Y*. Based on the global gene profile, the *ALPI* gene was selected as one of the possible candidates as the unique gene associated with C3 deficiency-induced constipation. The upregulation of *ALPI* expression by acetate treatment improved the key symptoms of constipation in epithelial cells and C3 KO mice with C3 deficiency. Taken together, our results suggest that *ALPI* can be considered a novel gene unique to constipation associated with C3 deficiency. However, this study had some limitations in that it did not confirm the expression level of all the differential genes using RT-qPCR because of the large number of genes. Moreover, the lack of analyses of the molecular mechanism of the effects of C3 deficiency-induced constipation in the transcriptional regulation of colon-expressed genes should be considered a drawback of this study. Also, further research will be needed to evaluate whether the exogenous administration of the protein encoded by the *ALPI* gene can be considered a treatment option to improve the symptoms of constipation in C3 KO mice.

## Figures and Tables

**Table 1:** Selection of the differentially expressed genes in the colon of C3 KO mice.

Categories	Number of Transcripts	Fold Change	
Total	41,345		
Filtered gene	33,793		
┃Fold change┃≥ 1.5 and raw. *p* < 0.05	2529	Up	1237
		Down	1292
┃Fold change┃≥ 2.0 and raw. *p* < 0.05	455	Up	226
		Down	229
┃Fold change┃≥ 3.0 and raw. *p* < 0.05	110	Up	38
		Down	72

**Table 2:** Selection of the differentially expressed genes in the colon of C3 KO mice.

Category Term	Count
**Upregulated genes**	
Biological regulation	13
Cell periphery	11
Cellular anatomical entity	5
Extracellular region	5
G protein-coupled receptor signaling pathway	1
Membrane	5
Molecular transducer activity	16
Molecular function	2
Multicellular organismal process	1
Odorant binding	6
Peptidase activity	2
Response to chemical	21
Response to stimulus	1
System process	6
tRNA dihydrouridine synthase activity	1
Immunoglobulin chain	30
Predicted gene	84
Blank	16
**Downregulated genes**	
Biological regulation	8
Cation binding	1
Cell periphery	21
Cellular anatomical entity	2
Extracellular region	18
G protein-coupled receptor signaling pathway	4
Hydrolase activity	3
Intermediate filament	1
Membrane	9
Molecular transducer activity	20
Molecular function	7
Multicellular organismal process	2
Organic acid binding	1
Response to chemical	22
Response to stimulus	6
System process	9
Keratin filament	1
Predicted gene	50
Immunoglobulin chain	23
Blank	21
**Total**	455

Each gene can be included in a category with duplicates.

**Table 3:** List of genes (by |FC| ≥ 3) upregulated by C3 deficiency-induced constipation.

Gene Symbol	Gene Name	Accession No.	Fold of Change	GO Category
Eif2s3y	eukaryotic translation initiation factor 2, subunit 3, structural gene Y-linked	NM_012011	29.68	Mus musculus eukaryotic translation initiation factor 2, subunit 3, structural gene Y-linked (Eif2s3y), mRNA
Uty	ubiquitously transcribed tetratricopeptide repeat gene, Y chromosome	NM_009484	24.65	Mus musculus ubiquitously transcribed tetratricopeptide repeat gene, Y chromosome (Uty), mRNA
Kdm5d	lysine (K)-specific demethylase 5D	NM_011419	19.14	Mus musculus lysine (K)-specific demethylase 5D (Kdm5d), mRNA
Igkv6-32	immunoglobulin kappa variable 6-32	OTTMUST00000132971	7.94	immunoglobulin kappa variable 6-32[gene_biotype:IG_gene transcript_biotype:IG_gene]
Ighv1-80	immunoglobulin heavy variable 1-80	OTTMUST00000131491	7.28	immunoglobulin heavy variable 1-80[gene_biotype:IG_gene transcript_biotype:IG_gene]
Igkv4-90	immunoglobulin kappa chain variable 4-90	OTTMUST00000132285	6.53	immunoglobulin kappa chain variable 4-90[gene_biotype:IG_gene transcript_biotype:IG_gene]
Igkv5-48	immunoglobulin kappa variable 5-48	OTTMUST00000132845	6.43	immunoglobulin kappa variable 5-48[gene_biotype:IG_gene transcript_biotype:IG_gene]
Ighv8-5	immunoglobulin heavy variable V8-5	OTTMUST00000131047	6.37	immunoglobulin heavy variable V8-5[gene_biotype:IG_gene transcript_biotype:IG_gene]
Olfr870	olfactory receptor 870	NM_146904	5.71	Mus musculus olfactory receptor 870 (Olfr870), mRNA
Ddx3y	DEAD (Asp-Glu-Ala-Asp) box polypeptide 3, Y-linked	NM_012008	5.65	Mus musculus DEAD (Asp-Glu-Ala-Asp) box polypeptide 3, Y-linked (Ddx3y), mRNA
Gm26194	predicted gene, 26194 [Source:MGI Symbol;Acc:MGI:5455971]	ENSMUST00000175036	5.57	predicted gene, 26194 [gene_biotype:miRNA transcript_biotype:miRNA]
Igkv8-30	immunoglobulin kappa chain variable 8-30	OTTMUST00000133006	5.42	immunoglobulin kappa chain variable 8-30[gene_biotype:IG_gene transcript_biotype:IG_gene]
Igkv8-21	immunoglobulin kappa variable 8-21	OTTMUST00000133067	4.82	immunoglobulin kappa variable 8-21[gene_biotype:IG_gene transcript_biotype:IG_gene]
Igkv3-7	immunoglobulin kappa variable 3-7	OTTMUST00000133340	4.71	immunoglobulin kappa variable 3-7[gene_biotype:IG_gene transcript_biotype:IG_gene]
Gm23479	predicted gene, 23479 [Source:MGI Symbol;Acc:MGI:5453256]	ENSMUST00000158876	4.20	predicted gene, 23479 [gene_biotype:snoRNA transcript_biotype:snoRNA]
Serpina1c	serine (or cysteine) peptidase inhibitor, clade A, member 1C	NM_009245	4.19	Mus musculus serine (or cysteine) peptidase inhibitor, clade A, member 1C (Serpina1c), mRNA
Hoxd13	homeobox D13	NM_008275	4.18	Mus musculus homeobox D13 (Hoxd13), mRNA
Gm25897	predicted gene, 25897 [Source:MGI Symbol;Acc:MGI:5455674]	ENSMUST00000083709	4.02	predicted gene, 25897 [gene_biotype:snRNA transcript_biotype:snRNA]
Gpx2	glutathione peroxidase 2	NM_030677	3.92	Mus musculus glutathione peroxidase 2 (Gpx2), mRNA
Ighm	immunoglobulin heavy constant mu	AF321953	3.79	Mus musculus BW2 24-13 immunoglobulin heavy chain mRNA, partial cds
Gm24032	predicted gene, 24032 [Source:MGI Symbol;Acc:MGI:5453809]	ENSMUST00000083932	3.79	predicted gene, 24032 [gene_biotype:snRNA transcript_biotype:snRNA]
Ighv4-1	immunoglobulin heavy variable 4-1	OTTMUST00000130463	3.50	immunoglobulin heavy variable 4-1[gene_biotype:IG_gene transcript_biotype:IG_gene]
Gm23280	predicted gene, 23280 [Source:MGI Symbol;Acc:MGI:5453057]	ENSMUST00000157905	3.35	predicted gene, 23280 [gene_biotype:snoRNA transcript_biotype:snoRNA]
Gm22149	predicted gene, 22149 [Source:MGI Symbol;Acc:MGI:5451926]	ENSMUST00000104442	3.33	predicted gene, 22149 [gene_biotype:snoRNA transcript_biotype:snoRNA]
Ifit3b	interferon-induced protein with tetratricopeptide repeats 3B	NM_001005858	3.31	Mus musculus interferon-induced protein with tetratricopeptide repeats 3B (Ifit3b), mRNA
		ENSMUST00000106852	3.31	predicted gene 10964 [gene_biotype:protein_coding transcript_biotype:protein_coding]
Gm22305	predicted gene, 22305 [Source:MGI Symbol;Acc:MGI:5452082]	ENSMUST00000082690	3.30	predicted gene, 22305 [gene_biotype:snRNA transcript_biotype:snRNA]
Igkv9-120	immunoglobulin kappa chain variable 9-120	OTTMUST00000131795	3.23	immunoglobulin kappa chain variable 9-120[gene_biotype:IG_gene transcript_biotype:IG_gene]
Klra9	killer cell lectin-like receptor subfamily A, member 9	NM_010651	3.19	Mus musculus killer cell lectin-like receptor subfamily A, member 9 (Klra9), mRNA
1700054O19Rik	RIKEN cDNA 1700054O19 gene	XR_383299	3.17	PREDICTED: Mus musculus RIKEN cDNA 1700054O19 gene (1700054O19Rik), misc_RNA
2200002J24Rik	RIKEN cDNA 2200002J24 gene	XM_006540347	3.15	PREDICTED: Mus musculus RIKEN cDNA 2200002J24 gene (2200002J24Rik), transcript variant X1, mRNA
Ighm	immunoglobulin heavy constant mu	AF045497	3.09	Mus musculus dC10 anti-poly(dC) monoclonal antibody heavy chain variable region, (IgH) mRNA, partial cds
Gm22521	predicted gene, 22521 [Source:MGI Symbol;Acc:MGI:5452298]	ENSMUST00000158272	3.08	predicted gene, 22521 [gene_biotype:snoRNA transcript_biotype:snoRNA]
Gm23626	predicted gene, 23626 [Source:MGI Symbol;Acc:MGI:5453403]	ENSMUST00000082458	3.07	predicted gene, 23626 [gene_biotype:snoRNA transcript_biotype:snoRNA]
Gm26375	predicted gene, 26375 [Source:MGI Symbol;Acc:MGI:5456152]	ENSMUST00000101829	3.03	predicted gene, 26375 [gene_biotype:snRNA transcript_biotype:snRNA]
Gm24555	predicted gene, 24555 [Source:MGI Symbol;Acc:MGI:5454332]	ENSMUST00000104548	3.02	predicted gene, 24555 [gene_biotype:snoRNA transcript_biotype:snoRNA]
Olfr555	olfactory receptor 555	NM_147103	3.01	Mus musculus olfactory receptor 555 (Olfr555), mRNA

**Table 4:** List of genes (by |FC| ≥ 3) downregulated by C3 deficiency-induced constipation.

Gene Symbol	Gene Name	Accession No.	Fold of Change	GO Category
Pnlip	pancreatic lipase	NM_026925	−658.36	Mus musculus pancreatic lipase (Pnlip), mRNA.
Cel	carboxyl ester lipase	NM_009885	−476.60	Mus musculus carboxyl ester lipase (Cel), mRNA.
Cpb1	carboxypeptidase B1 (tissue)	NM_029706	−257.26	Mus musculus carboxypeptidase B1 (tissue) (Cpb1), mRNA.
Ctrl	chymotrypsin-like	NM_023182	−214.25	Mus musculus chymotrypsin-like (Ctrl), mRNA.
2210010C04Rik	RIKEN cDNA 2210010C04 gene	NM_023333	−208.33	Mus musculus RIKEN cDNA 2210010C04 gene (2210010C04Rik), mRNA.
Pnliprp1	pancreatic lipase-related protein 1	NM_018874	−205.18	Mus musculus pancreatic lipase-related protein 1 (Pnliprp1), mRNA.
Reg1	regenerating islet-derived 1	NM_009042	−187.41	Mus musculus regenerating islet-derived 1 (Reg1), mRNA.
Amy2a5	amylase 2a5	NM_001042711	−183.01	Mus musculus amylase 2a5 (Amy2a5), mRNA.
Amy2a5	amylase 2a5	NM_001042711	−167.12	Mus musculus amylase 2a5 (Amy2a5), mRNA.
Amy2a5	amylase 2a5	NM_001042711	−162.29	Mus musculus amylase 2a5 (Amy2a5), mRNA.
Amy2a5	amylase 2a5	NM_001042711	−162.29	Mus musculus amylase 2a5 (Amy2a5), mRNA.
Cpa1	carboxypeptidase A1, pancreatic	NM_025350	−158.70	Mus musculus carboxypeptidase A1, pancreatic (Cpa1), mRNA.
Rnase1	ribonuclease, RNase A family, 1 (pancreatic)	NM_011271	−146.66	Mus musculus ribonuclease, RNase A family, 1 (pancreatic) (Rnase1), mRNA.
Ctrb1	chymotrypsinogen B1	NM_025583	−126.96	Mus musculus chymotrypsinogen B1 (Ctrb1), mRNA.
Cela2a	chymotrypsin-like elastase family, member 2A	NM_007919	−114.78	Mus musculus chymotrypsin-like elastase family, member 2A (Cela2a), mRNA.
Prss2	protease, serine 2	NM_009430	−94.18	Mus musculus protease, serine 2 (Prss2), mRNA.
Xist	inactive X-specific transcripts	NR_001463	−92.79	Mus musculus inactive X-specific transcripts (Xist), transcript variant 1, long non-coding RNA.
Gm5409	predicted pseudogene 5409	NM_001003664	−74.02	Mus musculus predicted pseudogene 5409 (Gm5409), mRNA.
Gp2	glycoprotein 2 (zymogen granule membrane)	NM_025989	−66.26	Mus musculus glycoprotein 2 (zymogen granule membrane) (Gp2), mRNA.
Amy2b	amylase 2b	NM_001190403	−64.57	Mus musculus amylase 2b (Amy2b), transcript variant 1, mRNA.
Serpini2	serine (or cysteine) peptidase inhibitor, clade I, member 2	NM_026460	−63.69	Mus musculus serine (or cysteine) peptidase inhibitor, clade I, member 2 (Serpini2), mRNA.
Pnliprp2	pancreatic lipase-related protein 2	NM_011128	−55.74	Mus musculus pancreatic lipase-related protein 2 (Pnliprp2), mRNA.
Pdia2	protein disulfide isomerase-associated 2	NM_001081070	−51.56	Mus musculus protein disulfide isomerase-associated 2 (Pdia2), mRNA.
Pla2g1b	phospholipase A2, group IB, pancreas	NM_011107	−49.31	Mus musculus phospholipase A2, group IB, pancreas (Pla2g1b), mRNA.
Ctrc	chymotrypsin C (caldecrin)	NM_001033875	−47.07	Mus musculus chymotrypsin C (caldecrin) (Ctrc), mRNA.
Prss1	protease, serine 1 (trypsin 1)	NM_053243	−44.45	Mus musculus protease, serine 1 (trypsin 1) (Prss1), mRNA.
Clps	colipase, pancreatic	NM_025469	−42.88	Mus musculus colipase, pancreatic (Clps), mRNA.
Cela3b	chymotrypsin-like elastase family, member 3B	NM_026419	−42.82	Mus musculus chymotrypsin-like elastase family, member 3B (Cela3b), mRNA.
Try4	trypsin 4	NM_011646	−35.21	Mus musculus trypsin 4 (Try4), mRNA.
Cpa2	carboxypeptidase A2, pancreatic	NM_001024698	−32.13	Mus musculus carboxypeptidase A2, pancreatic (Cpa2), mRNA.
Olfr764-ps1	olfactory receptor 764, pseudogene 1	XM_887185	−27.97	PREDICTED: Mus musculus olfactory receptor 764 (Olfr764), mRNA.
Cuzd1	CUB and zona pellucida-like domains 1	NM_008411	−24.78	Mus musculus CUB and zona pellucida-like domains 1 (Cuzd1), mRNA.
Cckar	cholecystokinin A receptor	NM_009827	−12.71	Mus musculus cholecystokinin A receptor (Cckar), mRNA.
Tff2	trefoil factor 2 (spasmolytic protein 1)	NM_009363	−10.82	Mus musculus trefoil factor 2 (spasmolytic protein 1) (Tff2), mRNA.
Cela1	chymotrypsin-like elastase family, member 1	NM_033612	−9.61	Mus musculus chymotrypsin-like elastase family, member 1 (Cela1), mRNA.
Ang4	angiogenin, ribonuclease A family, member 4	NM_177544	−8.63	Mus musculus angiogenin, ribonuclease A family, member 4 (Ang4), mRNA.
Tmed6	transmembrane emp24 protein transport domain containing 6	NM_025458	−7.42	Mus musculus transmembrane emp24 protein transport domain containing 6 (Tmed6), mRNA.
Ighv1-67	immunoglobulin heavy variable V1-67	OTTMUST00000131282	−7.09	immunoglobulin heavy variable V1-67[gene_biotype:IG_gene transcript_biotype:IG_gene]
Reg3d	regenerating islet-derived 3 delta	NM_001161741	−6.90	Mus musculus regenerating islet-derived 3 delta (Reg3d), transcript variant 2, mRNA.
Sptssb	serine palmitoyltransferase, small subunit B	NM_001164210	−6.68	Mus musculus serine palmitoyltransferase, small subunit B (Sptssb), transcript variant 1, mRNA.
Vtn	vitronectin	NM_011707	−5.72	Mus musculus vitronectin (Vtn), mRNA.
Tmed11	transmembrane emp24 protein transport domain containing	NM_026109	−5.08	Mus musculus transmembrane emp24 protein transport domain containing (Tmed11), mRNA.
Igkv6-17	immunoglobulin kappa variable 6-17	OTTMUST00000133153	−5.08	immunoglobulin kappa variable 6-17[gene_biotype:IG_gene transcript_biotype:IG_gene]
Aqp12	aquaporin 12	NM_001159658	−4.75	Mus musculus aquaporin 12 (Aqp12), transcript variant 2, mRNA.
1810007D17Rik	RIKEN cDNA 1810007D17 gene	NR_038136	−4.75	Mus musculus RIKEN cDNA 1810007D17 gene (1810007D17Rik), long non-coding RNA.
Try5	trypsin 5	NM_001003405	−4.59	Mus musculus trypsin 5 (Try5), mRNA.
Plet1	placenta expressed transcript 1	NM_029639	−4.51	Mus musculus placenta expressed transcript 1 (Plet1), mRNA.
Try10	trypsin 10	NM_001038996	−4.38	Mus musculus trypsin 10 (Try10), mRNA.
B3gnt6	UDP-GlcNAc:betaGal beta-1,3-N-acetylglucosaminyltransferase 6 (core 3 synthase)	NM_001081167	−4.20	Mus musculus UDP-GlcNAc:betaGal beta-1,3-N-acetylglucosaminyltransferase 6 (core 3 synthase) (B3gnt6), mRNA.
Itln1	intelectin 1 (galactofuranose binding)	NM_010584	−4.04	Mus musculus intelectin 1 (galactofuranose binding) (Itln1), mRNA.
Ighg	Immunoglobulin heavy chain (gamma polypeptide)	AB097847	−3.89	Mus musculus mRNA for immunoglobulin gamma-2a heavy chain, complete cds, anti-malathion monoclonal antibody MLT2-23.
Igkv3-4	immunoglobulin kappa variable 3-4	OTTMUST00000133343	−3.82	immunoglobulin kappa variable 3-4[gene_biotype:IG_gene transcript_biotype:IG_gene]
		GENSCAN00000010106	−3.75	cdna:genscan chromosome:GRCm38:19:28745871:28759892:-1 transcript_biotype:protein_coding
Gm25720	predicted gene, 25720 [Source:MGI Symbol;Acc:MGI:5455497]	ENSMUST00000157503	−3.72	predicted gene, 25720 [gene_biotype:snRNA transcript_biotype:snRNA]
Gm26441	predicted gene, 26441 [Source:MGI Symbol;Acc:MGI:5456218]	ENSMUST00000082590	−3.54	predicted gene, 26441 [gene_biotype:snRNA transcript_biotype:snRNA]
Chst2	carbohydrate sulfotransferase 2	NM_018763	−3.24	Mus musculus carbohydrate sulfotransferase 2 (Chst2), mRNA.
Vmn2r57	vomeronasal 2, receptor 57	NM_177764	−3.22	Mus musculus vomeronasal 2, receptor 57 (Vmn2r57), mRNA.
Fabp2	fatty acid-binding protein 2, intestinal	NM_007980	−3.20	Mus musculus fatty acid-binding protein 2, intestinal (Fabp2), mRNA.
		NONMMUT000288	−3.20	Non-coding transcript identified by NONCODE
Isx	intestine-specific homeobox	NM_001294278	−3.15	Mus musculus intestine-specific homeobox (Isx), transcript variant 1, mRNA.
Klk1b27	kallikrein 1-related peptidase b27	NM_020268	−3.09	Mus musculus kallikrein 1-related peptidase b27 (Klk1b27), mRNA.
Psg25	pregnancy-specific glycoprotein 25	NM_054060	−3.06	Mus musculus pregnancy-specific glycoprotein 25 (Psg25), mRNA.
Gm24762	predicted gene, 24762 [Source:MGI Symbol;Acc:MGI:5454539]	ENSMUST00000083689	−3.02	predicted gene, 24762 [gene_biotype:snRNA transcript_biotype:snRNA]
Ero1lb	ERO1-like beta (S. cerevisiae)	NM_026184	−3.02	Mus musculus ERO1-like beta (S. cerevisiae) (Ero1lb), mRNA.

## Data Availability

The microarray datasets produced in this study are available in the GEO database (http://www.ncbi.nlm.nih.gov/geo) under accession number GSE261184. Data are openly available in a public repository that issues datasets with DOIs.

## Supplementary Materials

The following supporting information can be downloaded at: https://www.mdpi.com/article/10.3390/ijms25179530/s1.
