# Supplementary material for: Novel Role of the ALPI Gene Associated with Constipation Caused by Complement Component 3 Deficiency
Source: Int J Mol Sci. 2024 Sep 2;25(17):9530. doi: 10.3390/ijms25179530 (PMC11395586; doi:10.3390/ijms25179530)
Supplement: Supplementary file 1 [file ijms-25-09530-s001.zip › ijms-3040949-supplementary.pdf]

International Journal of
Molecular Sciences

MDPI

Supplement Figure

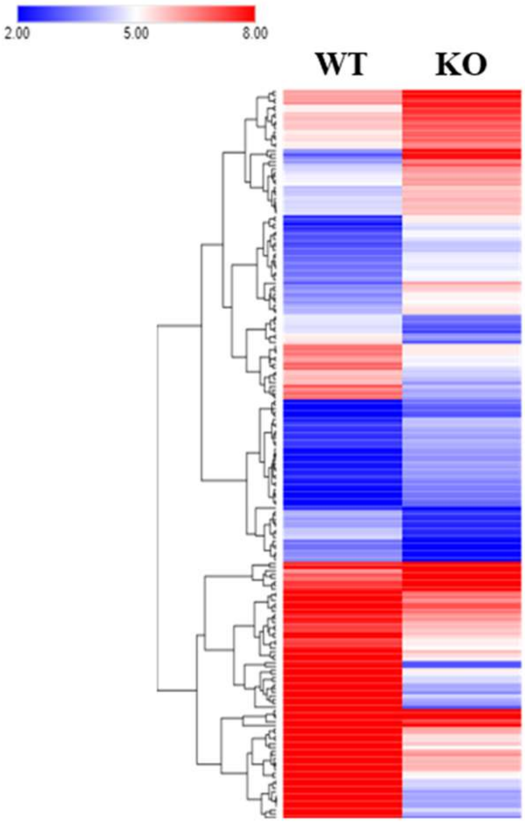

Supplement Figure S1. Microarray heat map and clustering based on hierarchy for the top 455 gene expression data (|FC| ≥ 2) from the datasets. Upregulated genes are represented in red, and downregulated genes are represented in blue. Abbreviation: FC, Fold change.

Supplement Table

Supplement Table S1. List of genes (by 3>|FC|≥2) upregulated by C3 deficiency-induced constipation

Gene Symbol	Gene Name	Accession No.	Fold of change	GO category
Gm23111	predicted gene, 23111 [Source:MGI Symbol;Acc:MGI:5452888]	ENSMUST00000122652	2.96	predicted gene, 23111 [gene_biotype:snRNA transcript_biotype:snRNA]
Gm25495	predicted gene, 25495 [Source:MGI Symbol;Acc:MGI:5455272]	ENSMUST00000104431	2.96	predicted gene, 25495 [gene_biotype:snoRNA transcript_biotype:snoRNA]
Gm26251	predicted gene, 26251 [Source:MGI Symbol;Acc:MGI:5456028]	ENSMUST00000122498	2.94	predicted gene, 26251 [gene_biotype:snoRNA transcript_biotype:snoRNA]
Cyp4a10	cytochrome P450, family 4, subfamily a, polypeptide 10	NM_010011	2.94	Mus musculus cytochrome P450, family 4, subfamily a, polypeptide 10 (Cyp4a10), mRNA.
		ENSMUST00000098716	2.94	predicted gene 10663 [gene_biotype:protein_coding transcript_biotype:protein_coding]
Gm22938	predicted gene, 22938 [Source:MGI Symbol;Acc:MGI:5452715]	ENSMUST00000103956	2.94	predicted gene, 22938 [gene_biotype:snoRNA transcript_biotype:snoRNA]
Alb	albumin	NM_009654	2.92	Mus musculus albumin (Alb), mRNA.
Gm5174	predicted gene 5174	XM_006514382	2.91	PREDICTED: Mus musculus predicted gene 5174 (Gm5174), transcript variant X2, mRNA.
Olfr110	olfactory receptor 110	NM_146328	2.85	Mus musculus olfactory receptor 110 (Olfr110), mRNA.
5033404E19Rik	NSA2 ribosome biogenesis homolog pseudogene	NR_033600	2.85	Mus musculus RIKEN cDNA 5033404E19 gene (5033404E19Rik), non-coding RNA.
Iglv3	immunoglobulin lambda variable 3	OTTMUST00000133490	2.81	immunoglobulin lambda variable 3[gene_biotype:IG_gene transcript_biotype:IG_gene]
Clec2j	C-type lectin domain family 2, member J	XM_006506842	2.81	PREDICTED: Mus musculus C-type lectin domain family 2, member J (Clec2j), mRNA.
Scel	sciellin	NM_022886	2.79	Mus musculus sciellin (Scel), mRNA.
Gm4759	GTPase, very large interferon inducible 1 pseudogene	NR_003967	2.77	Mus musculus predicted gene 4759 (Gm4759), non-coding RNA.
Gm24202	predicted gene, 24202 [Source:MGI Symbol;Acc:MGI:5453979]	ENSMUST00000082948	2.76	predicted gene, 24202 [gene_biotype:snRNA transcript_biotype:snRNA]
Cldn4	claudin 4	NM_009903	2.74	Mus musculus claudin 4 (Cldn4), mRNA.
Slc30a10	solute carrier family 30, member 10	NM_001033286	2.74	Mus musculus solute carrier family 30, member 10 (Slc30a10), mRNA.

Ighv8-8	immunoglobulin heavy variable 8-8	OTTMUST00000131094	2.73	immunoglobulin heavy variable 8-8[gene_biotype:IG_gene transcript_biotype:IG_gene]
Gm22573	predicted gene, 22573 [Source:MGI Symbol;Acc:MGI:5452350]	ENSMUST00000082982	2.73	predicted gene, 22573 [gene_biotype:snRNA transcript_biotype:snRNA]
Gm25524	predicted gene, 25524 [Source:MGI Symbol;Acc:MGI:5455301]	ENSMUST00000101821	2.72	predicted gene, 25524 [gene_biotype:snRNA transcript_biotype:snRNA]
Ighv1-81	immunoglobulin heavy variable 1-81	OTTMUST00000131492	2.66	immunoglobulin heavy variable 1-81[gene_biotype:IG_gene transcript_biotype:IG_gene]
4930553J12Rik	RIKEN cDNA 4930553J12 gene	XR_105792	2.64	PREDICTED: Mus musculus RIKEN cDNA 4930553J12 gene (4930553J12Rik), misc_RNA.
Rpp40	ribonuclease P 40 subunit	NM_145938	2.63	Mus musculus ribonuclease P 40 subunit (Rpp40), mRNA.
Gm25663	predicted gene, 25663 [Source:MGI Symbol;Acc:MGI:5455440]	ENSMUST00000157698	2.63	predicted gene, 25663 [gene_biotype:snoRNA transcript_biotype:snoRNA]
Igkv3-12	immunoglobulin kappa variable 3-12	OTTMUST00000133325	2.62	immunoglobulin kappa variable 3-12[gene_biotype:IG_gene transcript_biotype:IG_gene]
Gm25431	predicted gene, 25431 [Source:MGI Symbol;Acc:MGI:5455208]	ENSMUST00000101978	2.62	predicted gene, 25431 [gene_biotype:snRNA transcript_biotype:snRNA]
Mirlet7f-1	microRNA let7f-1	NR_029731	2.61	Mus musculus microRNA let7f-1 (Mirlet7f-1), microRNA.
Lilr4b	leukocyte immunoglobulin-like receptor, subfamily B, member 4B	NM_001291892	2.60	Mus musculus leukocyte immunoglobulin-like receptor, subfamily B, member 4B (Lilr4b), transcript variant 2, mRNA.
n-R5s29	nuclear encoded rRNA 5S 29 [Source:MGI Symbol;Acc:MGI:4421874]	ENSMUST00000082836	2.60	nuclear encoded rRNA 5S 29 [gene_biotype:rRNA transcript_biotype:rRNA]
Gm24880	predicted gene, 24880 [Source:MGI Symbol;Acc:MGI:5454657]	ENSMUST00000158776	2.59	predicted gene, 24880 [gene_biotype:rRNA transcript_biotype:rRNA]
Ighv7-3	immunoglobulin heavy variable 7-3	AF045506	2.59	Mus musculus 9E10 monoclonal antibody heavy chain variable region, (IgH) mRNA, partial cds.
Gm23014	predicted gene, 23014 [Source:MGI Symbol;Acc:MGI:5452791]	ENSMUST00000104144	2.59	predicted gene, 23014 [gene_biotype:snoRNA transcript_biotype:snoRNA]
Pzp	pregnancy zone protein	NM_007376	2.55	Mus musculus pregnancy zone protein (Pzp), mRNA.
Gm22005	predicted gene, 22005 [Source:MGI Symbol;Acc:MGI:5451782]	ENSMUST00000082795	2.54	predicted gene, 22005 [gene_biotype:snRNA transcript_biotype:snRNA]

Gm3543	predicted gene 3543 [Source:MGI Symbol;Acc:MGI:3781720]	ENSMUST00000111870	2.54	predicted gene 3543 [gene_biotype:protein_coding transcript_biotype:protein_coding]
Ms4a12	membrane-spanning 4-domains, subfamily A, member 12	XM_006527505	2.53	PREDICTED: Mus musculus membrane-spanning 4-domains, subfamily A, member 12 (Ms4a12), transcript variant X1, mRNA.
Olfr1140	olfactory receptor 1140	NM_146642	2.52	Mus musculus olfactory receptor 1140 (Olfr1140), mRNA.
Hist1h4d	histone cluster 1, H4d	OTTMUST00000001149	2.52	histone cluster 1, H4d[gene_biotype:protein_coding transcript_biotype:protein_coding]
4930596I21Rik	RIKEN cDNA 4930596I21 gene	NR_108103	2.51	Mus musculus RIKEN cDNA 4930596I21 gene (4930596I21Rik), long non-coding RNA.
Igkv14-111	immunoglobulin kappa variable 14-111	OTTMUST00000131890	2.51	immunoglobulin kappa variable 14-111[gene_biotype:IG_gene transcript_biotype:IG_gene]
Gm24258	predicted gene, 24258 [Source:MGI Symbol;Acc:MGI:5454035]	ENSMUST00000083739	2.50	predicted gene, 24258 [gene_biotype:snoRNA transcript_biotype:snoRNA]
Igkv8-24	immunoglobulin kappa chain variable 8-24	OTTMUST00000133030	2.47	immunoglobulin kappa chain variable 8-24[gene_biotype:IG_gene transcript_biotype:IG_gene]
Gm25764	predicted gene, 25764 [Source:MGI Symbol;Acc:MGI:5455541]	ENSMUST00000104092	2.47	predicted gene, 25764 [gene_biotype:snoRNA transcript_biotype:snoRNA]
Gm22764	predicted gene, 22764 [Source:MGI Symbol;Acc:MGI:5452541]	ENSMUST00000158370	2.46	predicted gene, 22764 [gene_biotype:snRNA transcript_biotype:snRNA]
Ms4a1	membrane-spanning 4-domains, subfamily A, member 1	NM_007641	2.46	Mus musculus membrane-spanning 4-domains, subfamily A, member 1 (Ms4a1), mRNA.
Igkv12-46	immunoglobulin kappa variable 12-46	OTTMUST00000132847	2.46	immunoglobulin kappa variable 12-46[gene_biotype:IG_gene transcript_biotype:IG_gene]
		NONMMUT051679	2.45	Non-coding transcript identified by NONCODE
		NONMMUT014107	2.44	Non-coding transcript identified by NONCODE
Gm11115	predicted gene 11115 [Source:MGI Symbol;Acc:MGI:3779367]	ENSMUST00000113255	2.43	predicted gene 11115 [gene_biotype:protein_coding transcript_biotype:protein_coding]
n-R5s218	nuclear encoded rRNA 5S 218 [Source:MGI Symbol;Acc:MGI:4422083]	ENSMUST00000122583	2.43	nuclear encoded rRNA 5S 218 [gene_biotype:rRNA transcript_biotype:rRNA]
		NONMMUT049198	2.41	Non-coding transcript identified by NONCODE: Antisense

Desi2	desumoylating isopeptidase 2	ENSMUST00000027783	2.41	desumoylating isopeptidase 2 [gene_biotype:protein_coding transcript_biotype:protein_coding]
Fgg	fibrinogen gamma chain	NM_133862	2.39	Mus musculus fibrinogen gamma chain (Fgg), mRNA.
Mup3	major urinary protein 3	NM_001039544	2.38	Mus musculus major urinary protein 3 (Mup3), mRNA.
Gm23561	predicted gene, 23561 [Source:MGI Symbol;Acc:MGI:5453338]	ENSMUST00000179812	2.37	predicted gene, 23561 [gene_biotype:snoRNA transcript_biotype:snoRNA]
		GENSCAN00000031592	2.36	cdna:genscan chromosome:GRCm38:19:16022373:16025720:1 transcript_biotype:protein_coding
Gm23237	predicted gene, 23237 [Source:MGI Symbol;Acc:MGI:5453014]	ENSMUST00000104639	2.36	predicted gene, 23237 [gene_biotype:miRNA transcript_biotype:miRNA]
Gm25406	predicted gene, 25406 [Source:MGI Symbol;Acc:MGI:5455183]	ENSMUST00000157599	2.35	predicted gene, 25406 [gene_biotype:snoRNA transcript_biotype:snoRNA]
Ighv1-77	immunoglobulin heavy variable 1-77	OTTMUST00000131410	2.34	immunoglobulin heavy variable 1-77[gene_biotype:IG_gene transcript_biotype:IG_gene]
		NONMMUT063737	2.34	Non-coding transcript identified by NONCODE: Linc
Gm23311	predicted gene, 23311 [Source:MGI Symbol;Acc:MGI:5453088]	ENSMUST00000104152	2.33	predicted gene, 23311 [gene_biotype:snoRNA transcript_biotype:snoRNA]
Gm6614	predicted gene 6614	NM_001081318	2.32	Mus musculus predicted gene 6614 (Gm6614), mRNA.
Gm23605	predicted gene, 23605 [Source:MGI Symbol;Acc:MGI:5453382]	ENSMUST00000157255	2.32	predicted gene, 23605 [gene_biotype:snoRNA transcript_biotype:snoRNA]
Igkv6-15	immunoglobulin kappa variable 6-15	OTTMUST00000133187	2.31	immunoglobulin kappa variable 6-15[gene_biotype:IG_gene transcript_biotype:IG_gene]
C7	complement component 7	NM_001243837	2.31	Mus musculus complement component 7 (C7), mRNA.
Pcdhb10	protocadherin beta 10	NM_053135	2.31	Mus musculus protocadherin beta 10 (Pcdhb10), mRNA.
Gm24261	predicted gene, 24261 [Source:MGI Symbol;Acc:MGI:5454038]	ENSMUST00000083744	2.30	predicted gene, 24261 [gene_biotype:snRNA transcript_biotype:snRNA]
Mir344d-3	microRNA 344d-3	NR_037209	2.30	Mus musculus microRNA 344d-3 (Mir344d-3), microRNA.
9230113P08Rik	RIKEN cDNA 9230113P08 gene	XM_006510720	2.30	PREDICTED: Mus musculus RIKEN cDNA 9230113P08 gene (9230113P08Rik), transcript variant X3, mRNA.
Gm25438	predicted gene, 25438 [Source:MGI Symbol;Acc:MGI:5455215]	ENSMUST00000158281	2.30	predicted gene, 25438 [gene_biotype:snoRNA transcript_biotype:snoRNA]

Gm23016	predicted gene, 23016 [Source:MGI Symbol;Acc:MGI:5452793]	ENSMUST00000158431	2.30	predicted gene, 23016 [gene_biotype:snRNA transcript_biotype:snRNA]
Gm12794	predicted gene 12794	NM_001085516	2.30	Mus musculus predicted gene 12794 (Gm12794), mRNA.
Mir466h	microRNA 466h	NR_030570	2.30	Mus musculus microRNA 466h (Mir466h), microRNA.
Gm5512	required for meiotic nuclear division 1 pseudogene	NR_002891	2.29	Mus musculus predicted gene 5512 (Gm5512), non-coding RNA.
Mir192	microRNA 192	NR_029720	2.29	Mus musculus microRNA 192 (Mir192), microRNA.
Olfr160	olfactory receptor 160	NM_030553	2.28	Mus musculus olfactory receptor 160 (Olfr160), mRNA.
n-R5s5	nuclear encoded rRNA 5S 5 [Source:MGI Symbol;Acc:MGI:4421739]	ENSMUST00000083134	2.28	nuclear encoded rRNA 5S 5 [gene_biotype:rRNA transcript_biotype:rRNA]
4732465J04Rik	RIKEN cDNA 4732465J04 gene	XR_380952	2.27	PREDICTED: Mus musculus RIKEN cDNA 4732465J04 gene (4732465J04Rik), misc_RNA.
Igkv17-127	immunoglobulin kappa variable 17-127	OTTMUST00000131759	2.27	immunoglobulin kappa variable 17-127[gene_biotype:IG_gene transcript_biotype:IG_gene]
Mir466i	microRNA 466i	NR_035412	2.27	Mus musculus microRNA 466i (Mir466i), microRNA.
Mir3087	microRNA 3087	NR_037270	2.27	Mus musculus microRNA 3087 (Mir3087), microRNA.
Gm26121	predicted gene, 26121 [Source:MGI Symbol;Acc:MGI:5455898]	ENSMUST00000083364	2.26	predicted gene, 26121 [gene_biotype:snRNA transcript_biotype:snRNA]
Igkv3-5	immunoglobulin kappa chain variable 3-5	OTTMUST00000133342	2.26	immunoglobulin kappa chain variable 3-5[gene_biotype:IG_gene transcript_biotype:IG_gene]
Gm24962	predicted gene, 24962 [Source:MGI Symbol;Acc:MGI:5454739]	ENSMUST00000157917	2.25	predicted gene, 24962 [gene_biotype:snRNA transcript_biotype:snRNA]
Ighm	immunoglobulin heavy constant mu	AF052834	2.25	Mus musculus hybridoma 12A1 immunoglobulin heavy chain mRNA, partial cds.
Gm24401	predicted gene, 24401 [Source:MGI Symbol;Acc:MGI:5454178]	ENSMUST00000157868	2.25	predicted gene, 24401 [gene_biotype:snoRNA transcript_biotype:snoRNA]
Gm24121	predicted gene, 24121 [Source:MGI Symbol;Acc:MGI:5453898]	ENSMUST00000102029	2.24	predicted gene, 24121 [gene_biotype:snRNA transcript_biotype:snRNA]
Gm25185	predicted gene, 25185 [Source:MGI Symbol;Acc:MGI:5454962]	ENSMUST00000157754	2.24	predicted gene, 25185 [gene_biotype:snoRNA transcript_biotype:snoRNA]
Gm22936	predicted gene, 22936 [Source:MGI Symbol;Acc:MGI:5452713]	ENSMUST00000103955	2.24	predicted gene, 22936 [gene_biotype:snoRNA transcript_biotype:snoRNA]

Oas1e	2-5 oligoadenylate synthetase 1E	NM_145210	2.23	Mus musculus 2-5 oligoadenylate synthetase 1E (Oas1e), mRNA.
Gm24894	predicted gene, 24894 [Source:MGI Symbol;Acc:MGI:5454671]	ENSMUST00000122744	2.23	predicted gene, 24894 [gene_biotype:snoRNA transcript_biotype:snoRNA]
Gm23026	predicted gene, 23026 [Source:MGI Symbol;Acc:MGI:5452803]	ENSMUST00000158772	2.23	predicted gene, 23026 [gene_biotype:snRNA transcript_biotype:snRNA]
2010111I01Rik	RIKEN cDNA 2010111I01 gene	NM_001289926	2.23	Mus musculus RIKEN cDNA 2010111I01 gene (2010111I01Rik), transcript variant 2, mRNA.
Trav6d-6	T cell receptor alpha variable 6D-6	M16676	2.22	Mus musculus T-cell receptor alpha-chain precursor, mRNA, partial cds.
Olfr1214	olfactory receptor 1214	NM_146897	2.22	Mus musculus olfactory receptor 1214 (Olfr1214), mRNA.
Gm26895	predicted gene, 26895 [Source:MGI Symbol;Acc:MGI:5477389]	ENSMUST00000181295	2.22	predicted gene, 26895 [gene_biotype:lincRNA transcript_biotype:lincRNA]
Gm21320	predicted gene, 21320	XM_003688783	2.22	PREDICTED: Mus musculus predicted gene, 21320 (Gm21320), transcript variant X1, mRNA.
		NONMMUT053515	2.22	Non-coding transcript identified by NONCODE
Gm22781	predicted gene, 22781 [Source:MGI Symbol;Acc:MGI:5452558]	ENSMUST00000104083	2.22	predicted gene, 22781 [gene_biotype:snoRNA transcript_biotype:snoRNA]
Hist1h3c	histone cluster 1, H3c	BC120800	2.22	Mus musculus histone cluster 1, H3c, mRNA (cDNA clone MGC:156037 IMAGE:40129723), complete cds.
Igkv4-79	immunoglobulin kappa variable 4-79	OTTMUST00000132413	2.21	immunoglobulin kappa variable 4-79[gene_biotype:IG_gene transcript_biotype:IG_gene]
Taar7d	trace amine-associated receptor 7D	NM_001010838	2.20	Mus musculus trace amine-associated receptor 7D (Taar7d), mRNA.
Mup7	major urinary protein 7	NM_001134675	2.19	Mus musculus major urinary protein 7 (Mup7), mRNA.
Klk1b8	kallikrein 1-related peptidase b8	NM_008457	2.19	Mus musculus kallikrein 1-related peptidase b8 (Klk1b8), mRNA.
Gm16185	predicted gene 16185	XR_379733	2.19	PREDICTED: Mus musculus predicted gene 16185 (Gm16185), ncRNA.
Mir30e	microRNA 30e	NR_029602	2.18	Mus musculus microRNA 30e (Mir30e), microRNA.
Mup19	major urinary protein 19	NM_001135127	2.18	Mus musculus major urinary protein 19 (Mup19), mRNA.
Igk-V1	immunoglobulin kappa chain variable 1 (V1)	AF154883	2.18	Mus musculus clone SIPC3282A immunoglobulin light chain variable region mRNA, partial cds.
Ahsg	alpha-2-HS-glycoprotein	NM_001276449	2.18	Mus musculus alpha-2-HS-glycoprotein (Ahsg), transcript variant 2, mRNA.

Mup14	major urinary protein 14	NM_001199999	2.17	Mus musculus major urinary protein 14 (Mup14), mRNA.
Mup18	major urinary protein 18	NM_001199333	2.17	Mus musculus major urinary protein 18 (Mup18), mRNA.
Mup13	major urinary protein 13	NM_001134674	2.17	Mus musculus major urinary protein 13 (Mup13), mRNA.
Olfr832	olfactory receptor 832	NM_001011824	2.17	Mus musculus olfactory receptor 832 (Olfr832), mRNA.
Olfr508	olfactory receptor 508	NM_146773	2.17	Mus musculus olfactory receptor 508 (Olfr508), mRNA.
Clca4b	chloride channel accessory 4B	NM_001033199	2.16	Mus musculus chloride channel accessory 4B (Clca4b), mRNA.
Mir20b	microRNA 20b	NR_030273	2.16	Mus musculus microRNA 20b (Mir20b), microRNA.
Gm24630	predicted gene, 24630 [Source:MGI Symbol;Acc:MGI:5454407]	ENSMUST00000122768	2.16	predicted gene, 24630 [gene_biotype:snRNA transcript_biotype:snRNA]
Gm25799	predicted gene, 25799 [Source:MGI Symbol;Acc:MGI:5455576]	ENSMUST00000082643	2.16	predicted gene, 25799 [gene_biotype:snRNA transcript_biotype:snRNA]
Gm26663	predicted gene, 26663	XR_881125	2.16	PREDICTED: Mus musculus uncharacterized LOC105247095 (LOC105247095), transcript variant X1, ncRNA.
		NONMMUT012306	2.15	Non-coding transcript identified by NONCODE
Gm26421	predicted gene, 26421 [Source:MGI Symbol;Acc:MGI:5456198]	ENSMUST00000104279	2.15	predicted gene, 26421 [gene_biotype:snoRNA transcript_biotype:snoRNA]
Hrg	histidine-rich glycoprotein	NM_053176	2.14	Mus musculus histidine-rich glycoprotein (Hrg), mRNA.
Mir200b	microRNA 200b	NR_029587	2.14	Mus musculus microRNA 200b (Mir200b), microRNA.
Igkv9-123	immunoglobulin kappa variable 9-123	OTTMUST00000131783	2.14	immunoglobulin kappa variable 9-123[gene_biotype:IG_gene transcript_biotype:IG_gene]
Fbxw24	F-box and WD-40 domain protein 24	NM_001013776	2.14	Mus musculus F-box and WD-40 domain protein 24 (Fbxw24), mRNA.
Gm24989	predicted gene, 24989 [Source:MGI Symbol;Acc:MGI:5454766]	ENSMUST00000104461	2.14	predicted gene, 24989 [gene_biotype:snoRNA transcript_biotype:snoRNA]
Gm24054	predicted gene, 24054 [Source:MGI Symbol;Acc:MGI:5453831]	ENSMUST00000158619	2.13	predicted gene, 24054 [gene_biotype:miRNA transcript_biotype:miRNA]
Olfr166	olfactory receptor 166	NM_147068	2.13	Mus musculus olfactory receptor 166 (Olfr166), mRNA.
Gm6525	ribosomal protein L36a pseudogene	NR_036654	2.12	Mus musculus predicted pseudogene 6525 (Gm6525), non-coding RNA.
Pax5	paired box 5	XM_006537673	2.12	PREDICTED: Mus musculus paired box 5 (Pax5), transcript variant X3, mRNA.

Gm11672	predicted gene 11672 [Source:MGI Symbol;Acc:MGI:3651419]	ENSMUST00000140380	2.12	predicted gene 11672 [gene_biotype:antisense transcript_biotype:antisense]
1700031A10Rik	RIKEN cDNA 1700031A10 gene	NR_045439	2.12	Mus musculus RIKEN cDNA 1700031A10 gene (1700031A10Rik), long non-coding RNA.
Gm20922	predicted gene, 20922 [Source:MGI Symbol;Acc:MGI:5434278]	ENSMUST00000186497	2.12	predicted gene, 20922 [gene_biotype:unprocessed_pseudogene transcript_biotype:unprocessed_pseudogene]
Olfr1090	olfactory receptor 1090	NM_146847	2.11	Mus musculus olfactory receptor 1090 (Olfr1090), mRNA.
Gm20506	predicted gene 20506	XR_385549	2.11	PREDICTED: Mus musculus predicted gene 20506 (Gm20506), transcript variant X6, ncRNA.
Gm25634	predicted gene, 25634 [Source:MGI Symbol;Acc:MGI:5455411]	ENSMUST00000082667	2.11	predicted gene, 25634 [gene_biotype:snRNA transcript_biotype:snRNA]
Gc	group specific component	NM_008096	2.11	Mus musculus group specific component (Gc), mRNA.
		NONMMUT066810	2.10	Non-coding transcript identified by NONCODE
Ifit1bl1	interferon induced protein with tetratricpeptide repeats 1B like 1	NM_001101605	2.10	Mus musculus interferon induced protein with tetratricpeptide repeats 1B like 1 (Ifit1bl1), transcript variant 2, mRNA.
Mir344e	microRNA 344e	NR_037284	2.10	Mus musculus microRNA 344e (Mir344e), microRNA.
		NONMMUT051567	2.10	Non-coding transcript identified by NONCODE: Sense No Exonic
Gm23698	predicted gene, 23698 [Source:MGI Symbol;Acc:MGI:5453475]	ENSMUST00000158836	2.10	predicted gene, 23698 [gene_biotype:snoRNA transcript_biotype:snoRNA]
Gm23169	predicted gene, 23169 [Source:MGI Symbol;Acc:MGI:5452946]	ENSMUST00000157157	2.10	predicted gene, 23169 [gene_biotype:snRNA transcript_biotype:snRNA]
Gm24078	predicted gene, 24078 [Source:MGI Symbol;Acc:MGI:5453855]	ENSMUST00000158184	2.10	predicted gene, 24078 [gene_biotype:miRNA transcript_biotype:miRNA]
Ifi44l	interferon-induced protein 44 like	OTTMUST00000133608	2.09	interferon-induced protein 44 like[gene_biotype:protein_coding transcript_biotype:protein_coding]
Mir301b	microRNA 301b	NR_030415	2.09	Mus musculus microRNA 301b (Mir301b), microRNA.
Klri1	killer cell lectin-like receptor family I member 1	NM_001012520	2.08	Mus musculus killer cell lectin-like receptor family I member 1 (Klri1), mRNA.
E330012B07Rik	RIKEN cDNA E330012B07 gene	NR_033640	2.08	Mus musculus RIKEN cDNA E330012B07 gene (E330012B07Rik), long non-coding RNA.

4930519F16Rik	RIKEN cDNA 4930519F16 gene	NM_029170	2.08	Mus musculus RIKEN cDNA 4930519F16 gene (4930519F16Rik), mRNA.
		NONMMUT067797	2.08	Non-coding transcript identified by NONCODE
Gm24141	predicted gene, 24141 [Source:MGI Symbol;Acc:MGI:5453918]	ENSMUST00000122776	2.08	predicted gene, 24141 [gene_biotype:snRNA transcript_biotype:snRNA]
Gm23321	predicted gene, 23321 [Source:MGI Symbol;Acc:MGI:5453098]	ENSMUST00000104465	2.08	predicted gene, 23321 [gene_biotype:snoRNA transcript_biotype:snoRNA]
Gm25921	predicted gene, 25921 [Source:MGI Symbol;Acc:MGI:5455698]	ENSMUST00000158305	2.08	predicted gene, 25921 [gene_biotype:snoRNA transcript_biotype:snoRNA]
Gm22740	predicted gene, 22740 [Source:MGI Symbol;Acc:MGI:5452517]	ENSMUST00000083439	2.08	predicted gene, 22740 [gene_biotype:snRNA transcript_biotype:snRNA]
Zfp942	zinc finger protein 942	NM_001199048	2.07	Mus musculus zinc finger protein 942 (Zfp942), mRNA.
Fam198b	family with sequence similarity 198, member B	NM_133187	2.07	Mus musculus family with sequence similarity 198, member B (Fam198b), mRNA.
Gm23622	predicted gene, 23622 [Source:MGI Symbol;Acc:MGI:5453399]	ENSMUST00000082460	2.06	predicted gene, 23622 [gene_biotype:snRNA transcript_biotype:snRNA]
Edn1	endothelin 1	NM_010104	2.06	Mus musculus endothelin 1 (Edn1), mRNA.
Gm23274	predicted gene, 23274 [Source:MGI Symbol;Acc:MGI:5453051]	ENSMUST00000104056	2.06	predicted gene, 23274 [gene_biotype:snoRNA transcript_biotype:snoRNA]
Gm25916	predicted gene, 25916 [Source:MGI Symbol;Acc:MGI:5455693]	ENSMUST00000104263	2.06	predicted gene, 25916 [gene_biotype:snoRNA transcript_biotype:snoRNA]
Gm25753	predicted gene, 25753 [Source:MGI Symbol;Acc:MGI:5455530]	ENSMUST00000116792	2.06	predicted gene, 25753 [gene_biotype:miRNA transcript_biotype:miRNA]
Gm24053	predicted gene, 24053 [Source:MGI Symbol;Acc:MGI:5453830]	ENSMUST00000158620	2.05	predicted gene, 24053 [gene_biotype:miRNA transcript_biotype:miRNA]
Nxpe2	neurexophilin and PC-esterase domain family, member 2	NM_030069	2.05	Mus musculus neurexophilin and PC-esterase domain family, member 2 (Nxpe2), mRNA.
Vmn1r148	vomeronasal 1 receptor 148	NM_030736	2.04	Mus musculus vomeronasal 1 receptor 148 (Vmn1r148), mRNA.
Gm22287	predicted gene, 22287 [Source:MGI Symbol;Acc:MGI:5452064]	ENSMUST00000083197	2.04	predicted gene, 22287 [gene_biotype:snRNA transcript_biotype:snRNA]
Nudt10	nudix (nucleoside diphosphate linked moiety X)-type motif 10	NM_001031664	2.04	Mus musculus nudix (nucleoside diphosphate linked moiety X)-type motif 10 (Nudt10), mRNA.

Gm24075	predicted gene, 24075 [Source:MGI Symbol;Acc:MGI:5453852]	ENSMUST00000158175	2.04	predicted gene, 24075 [gene_biotype:snoRNA transcript_biotype:snoRNA]
Gm16206	predicted gene 16206	XR_867285	2.03	PREDICTED: Mus musculus predicted gene 16206 (Gm16206), ncRNA.
Khdc1c	KH domain containing 1C	ENSMUST00000070223	2.03	KH domain containing 1C [gene_biotype:protein_coding transcript_biotype:protein_coding]
Mir669m-1	microRNA 669m-1	NR_035474	2.03	Mus musculus microRNA 669m-1 (Mir669m-1), microRNA.
Gm5925	germ cell-less homolog 1 family pseudogene	NR_040410	2.03	Mus musculus predicted gene 5925 (Gm5925), non-coding RNA.
Dus4l	dihydrouridine synthase 4-like (S. cerevisiae)	NM_028002	2.03	Mus musculus dihydrouridine synthase 4-like (S. cerevisiae) (Dus4l), mRNA.
Ugt2b1	UDP glucuronosyltransferase 2 family, polypeptide B1	NM_152811	2.02	Mus musculus UDP glucuronosyltransferase 2 family, polypeptide B1 (Ugt2b1), mRNA.
Mirlet7c-2	microRNA let7c-2	NR_029729	2.02	Mus musculus microRNA let7c-2 (Mirlet7c-2), microRNA.
Mup2	major urinary protein 2	NM_001045550	2.01	Mus musculus major urinary protein 2 (Mup2), transcript variant 2, mRNA.
n-R5s77	nuclear encoded rRNA 5S 77 [Source:MGI Symbol;Acc:MGI:4421925]	ENSMUST00000083757	2.01	nuclear encoded rRNA 5S 77 [gene_biotype:rRNA transcript_biotype:rRNA]
LOC102635992	major allergen I polypeptide chain 1-like	XM_006540491	2.01	PREDICTED: Mus musculus major allergen I polypeptide chain 1-like (LOC102635992), mRNA.
Olfr749	olfactory receptor 749	NM_020288	2.01	Mus musculus olfactory receptor 749 (Olfr749), mRNA.
Gm13051	predicted gene 13051 [Source:MGI Symbol;Acc:MGI:3702694]	ENSMUST00000105730	2.01	predicted gene 13051 [gene_biotype:protein_coding transcript_biotype:protein_coding]
Ccl27a	chemokine (C-C motif) ligand 27A	NM_001164044	2.01	Mus musculus chemokine (C-C motif) ligand 27A (Ccl27a), transcript variant 3, mRNA.
Gm23462	predicted gene, 23462 [Source:MGI Symbol;Acc:MGI:5453239]	ENSMUST00000083264	2.00	predicted gene, 23462 [gene_biotype:snRNA transcript_biotype:snRNA]
Sycp1-ps1	synaptonemal complex protein 1, pseudogene 1	NR_024208	2.00	Mus musculus synaptonemal complex protein 1, pseudogene 1 (Sycp1-ps1), non-coding RNA.
Nxpe4	neurexophilin and PC-esterase domain family, member 4	NM_172921	2.00	Mus musculus neurexophilin and PC-esterase domain family, member 4 (Nxpe4), mRNA.
Gm25528	predicted gene, 25528 [Source:MGI Symbol;Acc:MGI:5455305]	ENSMUST00000180264	2.00	predicted gene, 25528 [gene_biotype:snoRNA transcript_biotype:snoRNA]

Supplement Table S2. List of genes (by 3>|FC|≥2) downregulated by C3 deficiency-induced constipation

Gene Symbol	Gene Name	Accession No.	Fold of change	GO category
Gm23806	predicted gene, 23806 [Source:MGI Symbol;Acc:MGI:5453583]	ENSMUST00000175157	-2.99	predicted gene, 23806 [gene_biotype:rRNA transcript_biotype:rRNA]
Igkv4-58	immunoglobulin kappa variable 4-58	OTTMUST00000132705	-2.98	immunoglobulin kappa variable 4-58[gene_biotype:IG_gene transcript_biotype:IG_gene]
Gabra4	gamma-aminobutyric acid (GABA) A receptor, subunit alpha 4	NM_010251	-2.95	Mus musculus gamma-aminobutyric acid (GABA) A receptor, subunit alpha 4 (Gabra4), mRNA.
Rnase2b	ribonuclease, RNase A family, 2B (liver, eosinophil-derived neurotoxin)	NM_019398	-2.93	Mus musculus ribonuclease, RNase A family, 2B (liver, eosinophil-derived neurotoxin) (Rnase2b), mRNA.
Gm22549	predicted gene, 22549 [Source:MGI Symbol;Acc:MGI:5452326]	ENSMUST00000178461	-2.89	predicted gene, 22549 [gene_biotype:snRNA transcript_biotype:snRNA]
Gm22939	predicted gene, 22939 [Source:MGI Symbol;Acc:MGI:5452716]	ENSMUST00000103958	-2.86	predicted gene, 22939 [gene_biotype:snoRNA transcript_biotype:snoRNA]
n-R5s166	nuclear encoded rRNA 5S 166 [Source:MGI Symbol;Acc:MGI:4422030]	ENSMUST00000082898	-2.85	nuclear encoded rRNA 5S 166 [gene_biotype:rRNA transcript_biotype:rRNA]
Gm10238	predicted pseudogene 10238 [Source:MGI Symbol;Acc:MGI:3641620]	ENSMUST00000089689	-2.79	predicted pseudogene 10238 [gene_biotype:unprocessed_pseudogene transcript_biotype:unprocessed_pseudogene]
Slc13a1	solute carrier family 13 (sodium/sulfate symporters), member 1	NM_019481	-2.77	Mus musculus solute carrier family 13 (sodium/sulfate symporters), member 1 (Slc13a1), mRNA.
Pah	phenylalanine hydroxylase	NM_008777	-2.77	Mus musculus phenylalanine hydroxylase (Pah), mRNA.
B4galnt1	beta-1,4-N-acetyl-galactosaminyl transferase 1	NM_001244617	-2.76	Mus musculus beta-1,4-N-acetyl-galactosaminyl transferase 1 (B4galnt1), transcript variant 3, mRNA.
Glp1r	glucagon-like peptide 1 receptor	NM_021332	-2.76	Mus musculus glucagon-like peptide 1 receptor (Glp1r), mRNA.
Ly6g6c	lymphocyte antigen 6 complex, locus G6C	NM_023463	-2.76	Mus musculus lymphocyte antigen 6 complex, locus G6C (Ly6g6c), mRNA.
Gm23523	predicted gene, 23523 [Source:MGI Symbol;Acc:MGI:5453300]	ENSMUST00000180190	-2.76	predicted gene, 23523 [gene_biotype:snRNA transcript_biotype:snRNA]
Gm24793	predicted gene, 24793 [Source:MGI Symbol;Acc:MGI:5454570]	ENSMUST00000104289	-2.75	predicted gene, 24793 [gene_biotype:snoRNA transcript_biotype:snoRNA]

Vmn2r16	vomeronasal 2, receptor 16	NM_001104627	-2.71	Mus musculus vomeronasal 2, receptor 16 (Vmn2r16), mRNA.
Gm5449	predicted pseudogene 5449 [Source:MGI Symbol;Acc:MGI:3643792]	ENSMUST00000081132	-2.71	predicted pseudogene 5449 [gene_biotype:protein_coding transcript_biotype:protein_coding]
Aldh1l2	aldehyde dehydrogenase 1 family, member L2	NM_153543	-2.70	Mus musculus aldehyde dehydrogenase 1 family, member L2 (Aldh1l2), mRNA.
Igkv12-38	immunoglobulin kappa chain variable 12-38	OTTMUST00000132920	-2.66	immunoglobulin kappa chain variable 12-38[gene_biotype:IG_gene transcript_biotype:IG_gene]
1810009J06Rik	RIKEN cDNA 1810009J06 gene	NM_023707	-2.64	Mus musculus RIKEN cDNA 1810009J06 gene (1810009J06Rik), mRNA.
Nucb2	nucleobindin 2	NM_001130479	-2.62	Mus musculus nucleobindin 2 (Nucb2), mRNA.
n-R5s183	nuclear encoded rRNA 5S 183 [Source:MGI Symbol;Acc:MGI:4422048]	ENSMUST00000083939	-2.61	nuclear encoded rRNA 5S 183 [gene_biotype:rRNA transcript_biotype:rRNA]
Capn6	calpain 6	NM_007603	-2.60	Mus musculus calpain 6 (Capn6), mRNA.
Olfr1375	olfactory receptor 1375	NM_146516	-2.58	Mus musculus olfactory receptor 1375 (Olfr1375), mRNA.
Trav6-3	T cell receptor alpha variable 6-3	OTTMUST00000035959	-2.58	T-cell receptor alpha, variable 6-3[gene_biotype:TR_gene transcript_biotype:TR_gene]
C3	complement component 3	NM_009778	-2.54	Mus musculus complement component 3 (C3), mRNA.
Vsig2	V-set and immunoglobulin domain containing 2	NM_020518	-2.53	Mus musculus V-set and immunoglobulin domain containing 2 (Vsig2), mRNA.
Igkv9-124	immunoglobulin kappa chain variable 9-124	OTTMUST00000131782	-2.53	immunoglobulin kappa chain variable 9-124[gene_biotype:IG_gene transcript_biotype:IG_gene]
		NONMMUT023848	-2.52	Non-coding transcript identified by NONCODE
		NONMMUT033438	-2.52	Non-coding transcript identified by NONCODE: Linc
Traj43	T cell receptor alpha joining 43	OTTMUST00000134525	-2.52	T cell receptor alpha joining 43[gene_biotype:TR_gene transcript_biotype:TR_gene]
Klf15	Kruppel-like factor 15	NM_023184	-2.52	Mus musculus Kruppel-like factor 15 (Klf15), mRNA.
Gm5388	predicted gene 5388 [Source:MGI Symbol;Acc:MGI:3647395]	ENSMUST00000120059	-2.49	predicted gene 5388 [gene_biotype:processed_pseudogene transcript_biotype:processed_pseudogene]

Ighv1-73	immunoglobulin heavy variable 1-73	OTTMUST00000131350	-2.47	immunoglobulin heavy variable 1-73[gene_biotype:IG_pseudogene transcript_biotype:IG_pseudogene]
Gm17615	predicted gene, 17615 [Source:MGI Symbol;Acc:MGI:4937249]	ENSMUST00000166105	-2.47	predicted gene, 17615 [gene_biotype:protein_coding transcript_biotype:protein_coding]
Gm11309	predicted gene 11309 [Source:MGI Symbol;Acc:MGI:3650169]	ENSMUST00000127108	-2.46	predicted gene 11309 [gene_biotype:antisense transcript_biotype:antisense]
Abca12	ATP-binding cassette, sub-family A (ABC1), member 12	NM_175210	-2.45	Mus musculus ATP-binding cassette, sub-family A (ABC1), member 12 (Abca12), mRNA.
Gm20444	predicted gene 20444 [Source:MGI Symbol;Acc:MGI:5141909]	ENSMUST00000174014	-2.45	predicted gene 20444 [gene_biotype:processed_transcript transcript_biotype:processed_transcript]
Myh6	myosin, heavy polypeptide 6, cardiac muscle, alpha	NM_001164171	-2.45	Mus musculus myosin, heavy polypeptide 6, cardiac muscle, alpha (Myh6), transcript variant 1, mRNA.
Tmem28	transmembrane protein 28	NM_001081283	-2.44	Mus musculus transmembrane protein 28 (Tmem28), mRNA.
Casc4	cancer susceptibility candidate 4	NM_001205369	-2.44	Mus musculus cancer susceptibility candidate 4 (Casc4), transcript variant 3, mRNA.
Krt17	keratin 17	NM_010663	-2.43	Mus musculus keratin 17 (Krt17), mRNA.
Luzp4	leucine zipper protein 4	NM_001114383	-2.43	Mus musculus leucine zipper protein 4 (Luzp4), mRNA.
Retnlb	resistin like beta	NM_023881	-2.43	Mus musculus resistin like beta (Retnlb), mRNA.
Gm26276	predicted gene, 26276 [Source:MGI Symbol;Acc:MGI:5456053]	ENSMUST00000157099	-2.42	predicted gene, 26276 [gene_biotype:snoRNA transcript_biotype:snoRNA]
Olfr503	olfactory receptor 503	NM_001011527	-2.42	Mus musculus olfactory receptor 503 (Olfr503), mRNA.
Vmn2r9	vomeronasal 2, receptor 9	NM_001104621	-2.41	Mus musculus vomeronasal 2, receptor 9 (Vmn2r9), mRNA.
n-R5s43	nuclear encoded rRNA 5S 43 [Source:MGI Symbol;Acc:MGI:4421888]	ENSMUST00000122739	-2.40	nuclear encoded rRNA 5S 43 [gene_biotype:rRNA transcript_biotype:rRNA]
		GENSCAN00000051494	-2.40	cdna:genscan chromosome:GRCm38:7:11610117:11613559:1 transcript_biotype:protein_coding
Gm24573	predicted gene, 24573 [Source:MGI Symbol;Acc:MGI:5454350]	ENSMUST00000175499	-2.39	predicted gene, 24573 [gene_biotype:rRNA transcript_biotype:rRNA]

Gm23752	predicted gene, 23752 [Source:MGI Symbol;Acc:MGI:5453529]	ENSMUST00000157584	-2.39	predicted gene, 23752 [gene_biotype:rRNA transcript_biotype:rRNA]
Gm25614	predicted gene, 25614 [Source:MGI Symbol;Acc:MGI:5455391]	ENSMUST00000104013	-2.38	predicted gene, 25614 [gene_biotype:snoRNA transcript_biotype:snoRNA]
Gm25460	predicted gene, 25460 [Source:MGI Symbol;Acc:MGI:5455237]	ENSMUST00000157954	-2.38	predicted gene, 25460 [gene_biotype:snoRNA transcript_biotype:snoRNA]
Iglc3	immunoglobulin lambda constant 3	OTTMUST00000132547	-2.38	immunoglobulin lambda constant 3[gene_biotype:IG_gene transcript_biotype:IG_gene]
Gm25834	predicted gene, 25834 [Source:MGI Symbol;Acc:MGI:5455611]	ENSMUST00000158916	-2.37	predicted gene, 25834 [gene_biotype:snRNA transcript_biotype:snRNA]
Vmn2r72	vomeronasal 2, receptor 72	NM_001105185	-2.35	Mus musculus vomeronasal 2, receptor 72 (Vmn2r72), mRNA.
		NONMMUT017874	-2.34	Non-coding transcript identified by NONCODE
Gm25308	predicted gene, 25308 [Source:MGI Symbol;Acc:MGI:5455085]	ENSMUST00000104171	-2.34	predicted gene, 25308 [gene_biotype:snRNA transcript_biotype:snRNA]
Derl3	Der1-like domain family, member 3	NM_024440	-2.34	Mus musculus Der1-like domain family, member 3 (Derl3), mRNA.
		NONMMUT018767	-2.33	Non-coding transcript identified by NONCODE
Gm23448	predicted gene, 23448 [Source:MGI Symbol;Acc:MGI:5453225]	ENSMUST00000083151	-2.33	predicted gene, 23448 [gene_biotype:snRNA transcript_biotype:snRNA]
LOC102640059	uncharacterized LOC102640059	XR_383335	-2.32	PREDICTED: Mus musculus uncharacterized LOC102640059 (LOC102640059), transcript variant X7, ncRNA.
		NONMMUT073033	-2.32	Non-coding transcript identified by NONCODE
5430401H09Rik	RIKEN cDNA 5430401H09 gene	XR_381430	-2.30	PREDICTED: Mus musculus RIKEN cDNA 5430401H09 gene (5430401H09Rik), transcript variant X2, misc_RNA.
Gm23534	predicted gene, 23534 [Source:MGI Symbol;Acc:MGI:5453311]	ENSMUST00000157539	-2.30	predicted gene, 23534 [gene_biotype:snoRNA transcript_biotype:snoRNA]
Usp2	ubiquitin specific peptidase 2	NM_016808	-2.29	Mus musculus ubiquitin specific peptidase 2 (Usp2), transcript variant 1, mRNA.
Amy1	amylase 1, salivary	NM_001110505	-2.28	Mus musculus amylase 1, salivary (Amy1), transcript variant 2, mRNA.

Gm5356	predicted pseudogene 5356 [Source:MGI Symbol;Acc:MGI:3643649]	ENSMUST00000093326	-2.27	predicted pseudogene 5356 [gene_biotype:processed_pseudogene transcript_biotype:processed_pseudogene]
Cela3a	chymotrypsin-like elastase family, member 3A	NM_001126318	-2.26	Mus musculus chymotrypsin-like elastase family, member 3A (Cela3a), mRNA.
Olfr1390	olfactory receptor 1390	NM_147065	-2.26	Mus musculus olfactory receptor 1390 (Olfr1390), mRNA.
Olfr1361	olfactory receptor 1361	NM_146541	-2.26	Mus musculus olfactory receptor 1361 (Olfr1361), mRNA.
Cyp2b23	cytochrome P450, family 2, subfamily b, polypeptide 23	NM_001081148	-2.25	Mus musculus cytochrome P450, family 2, subfamily b, polypeptide 23 (Cyp2b23), mRNA.
Gm13780	predicted gene 13780 [Source:MGI Symbol;Acc:MGI:3650707]	ENSMUST00000150482	-2.24	predicted gene 13780 [gene_biotype:lincRNA transcript_biotype:lincRNA]
Nlrp5	NLR family, pyrin domain containing 5	NM_001039143	-2.24	Mus musculus NLR family, pyrin domain containing 5 (Nlrp5), transcript variant 2, mRNA.
Gm24520	predicted gene, 24520 [Source:MGI Symbol;Acc:MGI:5454297]	ENSMUST00000179510	-2.24	predicted gene, 24520 [gene_biotype:miRNA transcript_biotype:miRNA]
Cml2	camello-like 2	NM_053096	-2.24	Mus musculus camello-like 2 (Cml2), mRNA.
Klk1b21	kallikrein 1-related peptidase b21	NM_010642	-2.24	Mus musculus kallikrein 1-related peptidase b21 (Klk1b21), mRNA.
Gm23335	predicted gene, 23335 [Source:MGI Symbol;Acc:MGI:5453112]	ENSMUST00000117021	-2.23	predicted gene, 23335 [gene_biotype:miRNA transcript_biotype:miRNA]
Igkv4-80	immunoglobulin kappa variable 4-80	OTTMUST00000132412	-2.23	immunoglobulin kappa variable 4-80[gene_biotype:IG_gene transcript_biotype:IG_gene]
Tex9	testis expressed gene 9	NM_009359	-2.23	Mus musculus testis expressed gene 9 (Tex9), mRNA.
Gm23616	predicted gene, 23616 [Source:MGI Symbol;Acc:MGI:5453393]	ENSMUST00000177797	-2.21	predicted gene, 23616 [gene_biotype:miRNA transcript_biotype:miRNA]
Gm22120	predicted gene, 22120 [Source:MGI Symbol;Acc:MGI:5451897]	ENSMUST00000175590	-2.21	predicted gene, 22120 [gene_biotype:miRNA transcript_biotype:miRNA]
Gm23790	predicted gene, 23790 [Source:MGI Symbol;Acc:MGI:5453567]	ENSMUST00000083013	-2.20	predicted gene, 23790 [gene_biotype:snRNA transcript_biotype:snRNA]
Mir26a-1	microRNA 26a-1	NR_029742	-2.20	Mus musculus microRNA 26a-1 (Mir26a-1), microRNA.
1810018F18Rik	RIKEN cDNA 1810018F18 gene	NR_038140	-2.20	Mus musculus RIKEN cDNA 1810018F18 gene (1810018F18Rik), long non-coding RNA.

Olfr1414	olfactory receptor 1414	NM_147039	-2.20	Mus musculus olfactory receptor 1414 (Olfr1414), mRNA.
Gm25627	predicted gene, 25627 [Source:MGI Symbol;Acc:MGI:5455404]	ENSMUST00000084003	-2.20	predicted gene, 25627 [gene_biotype:snRNA transcript_biotype:snRNA]
Lct	lactase	NM_001081078	-2.19	Mus musculus lactase (Lct), mRNA.
Gm22598	predicted gene, 22598 [Source:MGI Symbol;Acc:MGI:5452375]	ENSMUST00000157600	-2.19	predicted gene, 22598 [gene_biotype:snoRNA transcript_biotype:snoRNA]
Olfr854	olfactory receptor 854	NM_146522	-2.18	Mus musculus olfactory receptor 854 (Olfr854), mRNA.
Ighv1-63	immunoglobulin heavy variable V1-63	OTTMUST00000131236	-2.18	immunoglobulin heavy variable V1-63[gene_biotype:IG_gene transcript_biotype:IG_gene]
Gm26073	predicted gene, 26073 [Source:MGI Symbol;Acc:MGI:5455850]	ENSMUST00000179906	-2.18	predicted gene, 26073 [gene_biotype:miRNA transcript_biotype:miRNA]
Gm24720	predicted gene, 24720 [Source:MGI Symbol;Acc:MGI:5454497]	ENSMUST00000157518	-2.17	predicted gene, 24720 [gene_biotype:snRNA transcript_biotype:snRNA]
Olfr1099	olfactory receptor 1099	NM_146768	-2.17	Mus musculus olfactory receptor 1099 (Olfr1099), mRNA.
Gm25249	predicted gene, 25249 [Source:MGI Symbol;Acc:MGI:5455026]	ENSMUST00000175471	-2.17	predicted gene, 25249 [gene_biotype:miRNA transcript_biotype:miRNA]
Pilrb2	paired immunoglobin-like type 2 receptor beta 2	NM_001024932	-2.17	Mus musculus paired immunoglobin-like type 2 receptor beta 2 (Pilrb2), mRNA.
n-R5s176	nuclear encoded rRNA 5S 176 [Source:MGI Symbol;Acc:MGI:4422041]	ENSMUST00000082683	-2.16	nuclear encoded rRNA 5S 176 [gene_biotype:rRNA transcript_biotype:rRNA]
Gm23322	predicted gene, 23322 [Source:MGI Symbol;Acc:MGI:5453099]	ENSMUST00000104464	-2.16	predicted gene, 23322 [gene_biotype:snRNA transcript_biotype:snRNA]
Gm30132	predicted gene, 30132	XR_387921	-2.15	PREDICTED: Mus musculus predicted gene, 30132 (Gm30132), transcript variant X1, ncRNA.
		NONMMUT029950	-2.15	Non-coding transcript identified by NONCODE
Gm41	melanoma antigen, family B, 4 pseudogene	NR_036689	-2.15	Mus musculus predicted pseudogene 41 (Gm41), non-coding RNA.
Gm26184	predicted gene, 26184 [Source:MGI Symbol;Acc:MGI:5455961]	ENSMUST00000157686	-2.15	predicted gene, 26184 [gene_biotype:snoRNA transcript_biotype:snoRNA]
Hoxc13	homeobox C13	NM_010464	-2.15	Mus musculus homeobox C13 (Hoxc13), mRNA.

Trgj3	T cell receptor gamma joining 3	OTTMUST00000134774	-2.15	T cell receptor gamma joining 3[gene_biotype:TR_gene transcript_biotype:TR_gene]
Car1	carbonic anhydrase 1	NM_001083957	-2.15	Mus musculus carbonic anhydrase 1 (Car1), transcript variant 2, mRNA.
		NONMMUT018234	-2.14	Non-coding transcript identified by NONCODE
Gm6194	transmembrane protein 189 pseudogene	NR_033512	-2.14	Mus musculus predicted gene 6194 (Gm6194), non-coding RNA.
Gm24678	predicted gene, 24678 [Source:MGI Symbol;Acc:MGI:5454455]	ENSMUST00000158756	-2.14	predicted gene, 24678 [gene_biotype:snRNA transcript_biotype:snRNA]
Mir669h	microRNA 669h	NR_035418	-2.14	Mus musculus microRNA 669h (Mir669h), microRNA.
Gm23003	predicted gene, 23003 [Source:MGI Symbol;Acc:MGI:5452780]	ENSMUST00000179948	-2.14	predicted gene, 23003 [gene_biotype:snRNA transcript_biotype:snRNA]
Gm22013	predicted gene, 22013 [Source:MGI Symbol;Acc:MGI:5451790]	ENSMUST00000158788	-2.13	predicted gene, 22013 [gene_biotype:snRNA transcript_biotype:snRNA]
Pak6	p21 protein (Cdc42/Rac)-activated kinase 6	NM_001033254	-2.13	Mus musculus p21 protein (Cdc42/Rac)-activated kinase 6 (Pak6), transcript variant 1, mRNA.
Ccin	calicin	NM_001002787	-2.12	Mus musculus calicin (Ccin), mRNA.
n-R5s19	nuclear encoded rRNA 5S 19 [Source:MGI Symbol;Acc:MGI:4421753]	ENSMUST00000122793	-2.12	nuclear encoded rRNA 5S 19 [gene_biotype:rRNA transcript_biotype:rRNA]
D830046C22Rik	RIKEN cDNA D830046C22 gene	NR_033147	-2.12	Mus musculus RIKEN cDNA D830046C22 gene (D830046C22Rik), long non-coding RNA.
Gm2825	predicted gene 2825	NM_001168337	-2.11	Mus musculus predicted gene 2825 (Gm2825), mRNA.
Slc9a3	solute carrier family 9 (sodium/hydrogen exchanger), member 3	NM_001081060	-2.11	Mus musculus solute carrier family 9 (sodium/hydrogen exchanger), member 3 (Slc9a3), mRNA.
		NONMMUT053036	-2.10	Non-coding transcript identified by NONCODE: Antisense
Banf2os	barrier to autointegration factor 2, opposite strand	XR_866789	-2.10	PREDICTED: Mus musculus barrier to autointegration factor 2, opposite strand (Banf2os), transcript variant X2, ncRNA.
5430416O09Rik	RIKEN cDNA 5430416O09 gene	NR_033355	-2.08	Mus musculus RIKEN cDNA 5430416O09 gene (5430416O09Rik), long non-coding RNA.
Cpn1	carboxypeptidase N, polypeptide 1	NM_030703	-2.08	Mus musculus carboxypeptidase N, polypeptide 1 (Cpn1), mRNA.

Gm23314	predicted gene, 23314 [Source:MGI Symbol;Acc:MGI:5453091]	ENSMUST00000157218	-2.08	predicted gene, 23314 [gene_biotype:snRNA transcript_biotype:snRNA]
Dux	double homeobox	NM_001081954	-2.08	Mus musculus double homeobox (Dux), mRNA.
Gm22070	predicted gene, 22070 [Source:MGI Symbol;Acc:MGI:5451847]	ENSMUST00000178475	-2.07	predicted gene, 22070 [gene_biotype:snRNA transcript_biotype:snRNA]
Adra1d	adrenergic receptor, alpha 1d	NM_013460	-2.07	Mus musculus adrenergic receptor, alpha 1d (Adra1d), mRNA.
Gm22792	predicted gene, 22792 [Source:MGI Symbol;Acc:MGI:5452569]	ENSMUST00000157225	-2.07	predicted gene, 22792 [gene_biotype:snoRNA transcript_biotype:snoRNA]
Gm17079	predicted gene 17079	XR_874617	-2.07	PREDICTED: Mus musculus predicted gene 17079 (Gm17079), ncRNA.
Cntn5	contactin 5	NM_001033359	-2.07	Mus musculus contactin 5 (Cntn5), transcript variant 2, mRNA.
Alpi	alkaline phosphatase, intestinal	NM_001081082	-2.06	Mus musculus alkaline phosphatase, intestinal (Alpi), mRNA.
Krt31	keratin 31	NM_010659	-2.06	Mus musculus keratin 31 (Krt31), mRNA.
Nov	nephroblastoma overexpressed gene	NM_010930	-2.06	Mus musculus nephroblastoma overexpressed gene (Nov), mRNA.
Olfr45	olfactory receptor 45	NM_146963	-2.06	Mus musculus olfactory receptor 45 (Olfr45), mRNA.
1700044C05Rik	RIKEN cDNA 1700044C05 gene	NR_045624	-2.06	Mus musculus RIKEN cDNA 1700044C05 gene (1700044C05Rik), long non-coding RNA.
Stk-ps2	serine/threonine kinase 2	NR_037690	-2.06	Mus musculus serine/threonine kinase 2 (Stk-ps2), transcript variant 2, non-coding RNA.
Gm25937	predicted gene, 25937 [Source:MGI Symbol;Acc:MGI:5455714]	ENSMUST00000083912	-2.05	predicted gene, 25937 [gene_biotype:snRNA transcript_biotype:snRNA]
Gm25873	predicted gene, 25873 [Source:MGI Symbol;Acc:MGI:5455650]	ENSMUST00000180317	-2.05	predicted gene, 25873 [gene_biotype:snRNA transcript_biotype:snRNA]
		NONMMUT058433	-2.05	Non-coding transcript identified by NONCODE: Antisense
Neurod2	neurogenic differentiation 2	NM_010895	-2.04	Mus musculus neurogenic differentiation 2 (Neurod2), mRNA.
Fkbp11	FK506 binding protein 11	NM_024169	-2.04	Mus musculus FK506 binding protein 11 (Fkbp11), mRNA.
Gm3250	predicted gene 3250	XM_006514351	-2.04	PREDICTED: Mus musculus predicted gene 3250 (Gm3250), mRNA.
Gm25906	predicted gene, 25906 [Source:MGI Symbol;Acc:MGI:5455683]	ENSMUST00000158489	-2.03	predicted gene, 25906 [gene_biotype:snoRNA transcript_biotype:snoRNA]

Muc4	mucin 4	NM_080457	-2.03	Mus musculus mucin 4 (Muc4), mRNA.
Dmbt1	deleted in malignant brain tumors 1	NM_007769	-2.03	Mus musculus deleted in malignant brain tumors 1 (Dmbt1), mRNA.
Gm25589	predicted gene, 25589 [Source:MGI Symbol;Acc:MGI:5455366]	ENSMUST00000082502	-2.03	predicted gene, 25589 [gene_biotype:snRNA transcript_biotype:snRNA]
Asns	asparagine synthetase	NM_012055	-2.03	Mus musculus asparagine synthetase (Asns), mRNA.
Gm19554	predicted gene, 19554	XR_871169	-2.03	PREDICTED: Mus musculus predicted gene, 19554 (Gm19554), transcript variant X1, ncRNA.
Atp12a	ATPase, H+/K+ transporting, nongastric, alpha polypeptide	NM_138652	-2.02	Mus musculus ATPase, H+/K+ transporting, nongastric, alpha polypeptide (Atp12a), mRNA.
Gm22427	predicted gene, 22427 [Source:MGI Symbol;Acc:MGI:5452204]	ENSMUST00000103922	-2.02	predicted gene, 22427 [gene_biotype:miRNA transcript_biotype:miRNA]
Gm23251	predicted gene, 23251 [Source:MGI Symbol;Acc:MGI:5453028]	ENSMUST00000103773	-2.02	predicted gene, 23251 [gene_biotype:miRNA transcript_biotype:miRNA]
Zfp474	zinc finger protein 474	NM_025749	-2.02	Mus musculus zinc finger protein 474 (Zfp474), mRNA.
Olfr680-ps1	olfactory receptor 680, pseudogene 1	OTTMUST00000112019	-2.02	olfactory receptor 680, pseudogene 1[gene_biotype:unprocessed_pseudogene transcript_biotype:unprocessed_pseudogene]
Trbj1-7	T cell receptor beta joining 1-7	OTTMUST00000129890	-2.01	T cell receptor beta joining 1-7[gene_biotype:TR_gene transcript_biotype:TR_gene]
Taf1d	TATA box binding protein (Tbp)-associated factor, RNA polymerase I, D	XR_870541	-2.01	PREDICTED: Mus musculus TATA box binding protein (Tbp)-associated factor, RNA polymerase I, D (Taf1d), transcript variant X3, misc_RNA.
Gm24919	predicted gene, 24919 [Source:MGI Symbol;Acc:MGI:5454696]	ENSMUST00000083020	-2.00	predicted gene, 24919 [gene_biotype:snRNA transcript_biotype:snRNA]
Fkbp5	FK506 binding protein 5	NM_010220	-2.00	Mus musculus FK506 binding protein 5 (Fkbp5), mRNA.
Prss3	protease, serine 3	NM_011645	-2.00	Mus musculus protease, serine 3 (Prss3), mRNA.

Supplement Table S3. RT-qPCR primer sequence of target gene

Gene name	Sequence (from 5′ to 3′)
EDN1	
Forward	AAAAA ACCCA GAGGC GATCA
Reverse	TGCAA ATGGA ACTCA ATGCA A
RETNLB	
Forward	CCTGC ACTAG TGTCA CGTCT TCTG
Reverse	AAGCA CATCC AGTGA CAACC AT
NXPE4	
Forward	CACCA CCATT CCGAA TTGG
Reverse	AGCTG TCCAG TGAAA CCATC TCT
ALPI	
Forward	TGGCA ATGGT CCAGG TTATG A
Reverse	AACAC TCTGA GCTTC GGTGA CA
C-kit	
Forward	TGTTG CCTTC ACGGT TTTCC
Reverse	AACGA TCACT TCTTC CAGGT TCA
MUC1	
Forward	CGCCA GCCTT GAGTT TGTTT
Reverse	GAAGA AAGGA GCCCG AATGC
MUC2	
Forward	GCA CAT TCC TTC GCA TCT TAA A
Reverse	AAA GCA AAG AAT GGA ACA GAA CAG AAA CTC
KLF4	
Forward	GGTGC AGCTT GCAGC AGTAA
Reverse	AAGTC TAGGT CCAGG AGGTC GTT
AQP3	
Forward	GGTGG TCCTG GTCAT TGGAA
Reverse	AGTCA CGGGC AGGGT TGA
AQP8	
Forward	TCGCT GGCAG TCACA GTGA
Reverse	TCCAA ATAGC TGGGA GATCC A
β-actin	
Forward	TGGAA TCCTG TGGCA TCCAT GAAAC
Reverse	TAAAA CGCAG CTCAG TAACA GTCCG
